# Molecular basis for differential *Igk* versus *Igh* V(D)J joining mechanisms

**DOI:** 10.1038/s41586-024-07477-y

**Published:** 2024-05-29

**Authors:** Yiwen Zhang, Xiang Li, Zhaoqing Ba, Jiangman Lou, K. Elyse Gaertner, Tammie Zhu, Xin Lin, Adam Yongxin Ye, Frederick W. Alt, Hongli Hu

**Affiliations:** 1 Howard Hughes Medical Institute, Program in Cellular and Molecular Medicine, Boston Children’s Hospital, Boston, MA USA; 2 Department of Genetics, Harvard Medical School, Boston, MA USA; 3 Present Address: National Institute of Biological Sciences, Beijing, China; 4 Present Address: Copenhagen University, Copenhagen, Denmark; 5 Present Address: Georgetown University, Washington, DC USA

**Keywords:** Epigenetics in immune cells, Gene regulation, DNA recombination

## Abstract

In developing B cells, V(D)J recombination assembles exons encoding IgH and Igκ variable regions from hundreds of gene segments clustered across *Igh* and *Igk* loci. V, D and J gene segments are flanked by conserved recombination signal sequences (RSSs) that target RAG endonuclease^1^. RAG orchestrates *Igh* V(D)J recombination upon capturing a J_H_-RSS within the J_H_-RSS-based recombination centre^1–3^ (RC). J_H_-RSS orientation programmes RAG to scan upstream D- and V_H_-containing chromatin that is presented in a linear manner by cohesin-mediated loop extrusion^4–7^. During *Igh* scanning, RAG robustly utilizes only D-RSSs or V_H_-RSSs in convergent (deletional) orientation with J_H_-RSSs^4–7^. However, for Vκ-to-Jκ joining, RAG utilizes Vκ-RSSs from deletional- and inversional-oriented clusters^8^, inconsistent with linear scanning^2^. Here we characterize the Vκ-to-Jκ joining mechanism. *Igk* undergoes robust primary and secondary rearrangements^9,10^, which confounds scanning assays. We therefore engineered cells to undergo only primary Vκ-to-Jκ rearrangements and found that RAG scanning from the primary Jκ-RC terminates just 8 kb upstream within the CTCF-site-based *Sis* element^11^. Whereas *Sis* and the Jκ-RC barely interacted with the Vκ locus, the CTCF-site-based *Cer* element^12^ 4 kb upstream of *Sis* interacted with various loop extrusion impediments across the locus. Similar to V_H_ locus inversion^7^, DJ_H_ inversion abrogated V_H_-to-DJ_H_ joining; yet Vκ locus or Jκ inversion allowed robust Vκ-to-Jκ joining. Together, these experiments implicated loop extrusion in bringing Vκ segments near *Cer* for short-range diffusion-mediated capture by RC-based RAG. To identify key mechanistic elements for diffusional V(D)J recombination in *Igk* versus *Igh*, we assayed Vκ-to-J_H_ and D-to-Jκ rearrangements in hybrid *Igh–Igk* loci generated by targeted chromosomal translocations, and pinpointed remarkably strong Vκ and Jκ RSSs. Indeed, RSS replacements in hybrid or normal *Igk* and *Igh* loci confirmed the ability of *Igk*-RSSs to promote robust diffusional joining compared with *Igh-*RSSs. We propose that *Igk* evolved strong RSSs to mediate diffusional Vκ-to-Jκ joining, whereas *Igh* evolved weaker RSSs requisite for modulating V_H_ joining by RAG-scanning impediments.

## Abstract (web summary)

Experiments in mouse models, and in cell lines that only allow primary Vκ-to-Jκ rearrangements, enable characterization of the mechanisms of V(D)J recombination.

## Main

Bona fide RSSs flanking antigen receptor gene segments have a conserved palindromic heptamer with a consensus CACAGTG sequence and a less-conserved AT-rich nonamer separated by 12-bp or 23-bp spacers^1^ (denoted 12RSSs and 23RSSs, respectively). RAG endonuclease initiates V(D)J recombination by cleaving between the CAC of the heptamer and flanking coding sequences upon capturing complementary 12RSSs and 23RSSs in its two active sites, a property known as 12/23 restriction^1,13,14^. In the mouse *Igh*, more than 100 V_H_ segments lie within a 2.4 Mb distal portion followed downstream by multiple D segments and four J_H_ segments^2^. V_H_ segments have downstream 23RSSs, D segments have 12RSSs on both sides, and J_H_ segments have upstream 23RSSs^2^. Owing to 12/23 restriction, V_H_ segments cannot directly join to J_H_ segments. In progenitor B (pro-B) cells, joining of all D segments, except proximal DQ52, to J_H_ segments occurs via linear scanning during which RAG dominantly captures and utilizes downstream, deletional D-12RSSs owing to convergent orientation with J_H_-23RSSs^5^. As DQ52 lies within the *Igh*-RC, both of its RSSs access RAG by short-range diffusion, but the downstream DQ52-12RSS predominates owing to its superior strength^2,5,15^. The DJ_H_ intermediate and its upstream 12RSS form a RC for V_H_-to-DJ_H_ joining^2,3^; but the IGCR1 regulatory region just upstream of the D segments contains two CTCF-binding elements (CBEs) that substantially impede upstream RAG scanning^4,6,16^. Moreover, most D-proximal V_H_ segments have RSS-associated CBEs that impede RAG scanning and enhance their interaction with the DJ_H_-RC, increasing their utilization far beyond that provided by their RSSs alone^3^. To promote balanced V_H_ utilization, the activity of CBEs and other V_H_ locus scanning impediments is diminished in pro-B cells by developmental down-modulation of the WAPL cohesin-unloading factor^7,17^, enabling linear loop extrusion to directly present the entire V_H_ locus to the RAG-bound DJ_H_-RC^7^. Although RAG linearly scans the length of an inverted V_H_ locus, V_H_-to-DJ_H_ joining is nearly abrogated due to bona fide V_H_-RSSs no longer being in convergent orientation with the DJ_H_-RC RSS^7^.

## Primary Vκ-to-Jκ joining does not use RAG scanning

**Fig. 1:**
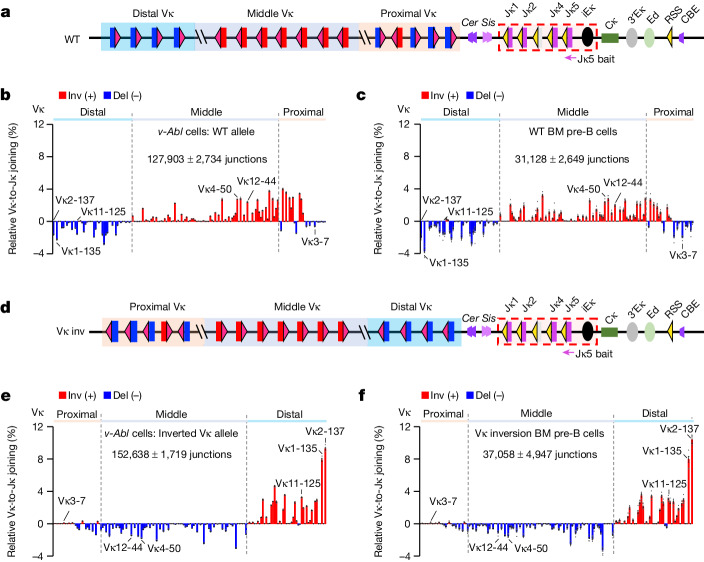
Vκ locus inversion maintains utilization of deletional and inversional Vκ segments in bone marrow pre-B cells and in *v-Abl* cells. **a**, Illustration of mouse *Igk* (not to scale). Relative location of proximal (orange shadow) and distal (blue shadow) mainly deletional-oriented Vκ segments and middle (grey shadow) mainly inversional-oriented Vκ segments. *Cer* and *Sis* lie downstream of the proximal Vκ; *Cer* upstream-oriented (purple trapezoids) and *Sis* downstream-oriented (pink trapezoids) CBEs are indicated. Four functional Jκ segments downstream of *Sis*, with the *Igk* intronic enhancer (iEκ), form the RC (dashed red rectangle). Further downstream, the Cκ, *Igk* enhancers, RSS and upstream-oriented CBE are indicated. Vκ segments are flanked by 12RSSs (red triangles) and Jκ segments by 23RSSs (yellow triangles). Vκ locus CBEs are shown in Fig. 2. WT, wild type. **b**,**c**, Relative utilization of individual Vκ segments on wild-type alleles in *v-Abl* cells (**b**) and bone marrow (BM) pre-B cells (**c**) baiting from Jκ5 (indicated in **a**). Inv, inversional joins; Del, deletional joins. Locations of selected Vκ segments are indicated—these features are retained in subsequent figures. Vκ usage patterns in **b**,**c** are highly similar (two-sided Pearson’s *r* = 0.88, *P* = 2.2 × 10^−53^). **d**, Illustration of inverted Vκ locus. **e**,**f**, Relative utilization of individual Vκ segments on inverted Vκ alleles in *v-Abl* cells (**e**) and bone marrow pre-B cells (**f**) assayed with Jκ5 bait. Vκ usage data in the inverted locus is shown in the inverted orientation. Vκ usage patterns in **e**,**f** are highly similar (two-sided Pearson’s *r* = 0.97, *P* = 5.5 × 10^−97^). Junction numbers are shown in each panel and in subsequent figures for comparison of absolute levels. Vκ utilization data are presented as mean ± s.e.m. from 4 (**b**,**e**) or 7 (**c**,**f**) biological repeats. Source Data

The distal 3 Mb of mouse *Igk* contains 103 functional Vκ segments associated with 12RSSs followed downstream by the *Igk*-RC that contains 4 functional Jκ segments with 23RSSs, allowing direct Vκ-to-Jκ joining^8^ (Fig. 1a). The *Cer* and *Sis* elements, each of which contain two CBEs and are located in the 13 kb interval between the most proximal Vκ and Jκ1 (Fig. 1a), functionally promote distal Vκ usage^11,12^. In precursor B (pre-B) cells, initial (primary) Vκ-to-Jκ rearrangements mostly utilize Jκ1^18^. Subsequently, the three functional downstream Jκ segments (Jκ2, Jκ4 and Jκ5) undergo secondary rearrangements with remaining upstream Vκ segments^18^. V(D)J recombination, which occurs strictly in the G1 phase of the cell cycle^19^, can be activated in G1-arrested Abelson murine leukaemia virus-transformed pro-B cell lines^20^ (hereafter referred to as ‘*v-Abl* cells’). For high-throughput genome-wide translocation sequencing-adapted V(D)J-sequencing (HTGTS-V(D)J-seq) assays^21^, we generated RAG-deficient *v-Abl* cells and ectopically introduced RAG upon G1 arrest. Although *v-Abl* cells undergo robust D-to-J_H_ rearrangements, they rarely exhibit V_H_-to-DJ_H_ rearrangements owing to high levels of WAPL^7^. Despite these high WAPL levels, *v-Abl* cells underwent robust Vκ-to-Jκ rearrangements with usage patterns of deletional- and inversional-oriented Vκ segments similar to those of normal bone marrow pre-B cells (Fig. 1b,c). Of note, bone marrow pre-B cells and *v-Abl* cells in which we inverted the Vκ locus (Fig. 1d) underwent very similar patterns of robust Vκ-to-Jκ rearrangements, with previously deletional-oriented Vκ segments rearranging by inversion and previously inversional-oriented Vκ segments rearranging by deletion (Fig. 1e,f). These results confirm that *Igk* utilizes a markedly different long-range V(D)J recombination mechanism to that of *Igh* and indicate that *v-Abl* lines are a faithful system for in depth analyses of this mechanism.

**Fig. 2:**
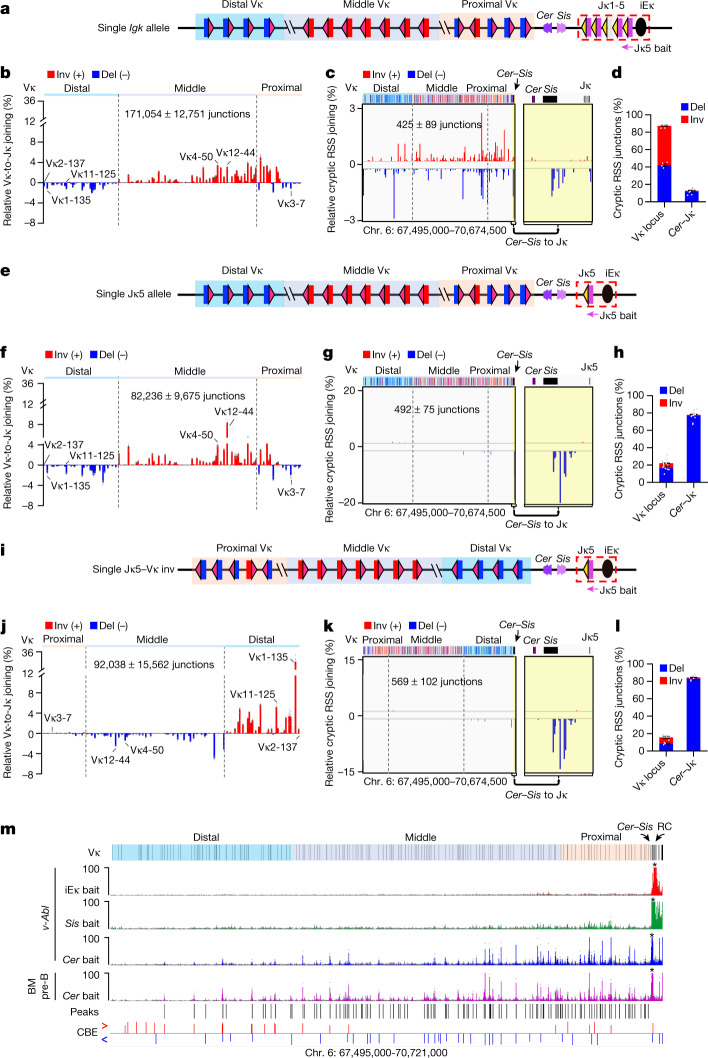
RAG scanning for primary *Igk* rearrangement is terminated within *Sis* while *Cer* interacts across the Vκ locus. **a**, Diagram of the single *Igk* allele *v-Abl* line. **b**, Relative utilization of individual Vκ segments in the single *Igk* allele line with Jκ5 bait. **c**, Percentage of pooled RAG off-target junctions in *Igk* locus from the single *Igk* allele line. The region between *Cer* and Jκ, highlighted in yellow, is enlarged on the right. **d**, Percentage of inversional and deletional cryptic RSS junctions within indicated Vκ locus (chromosome (chr.) 6:67,495,000–70,657,000) and *Cer*–Jκ regions (chr. 6:70,657,000–70,674,500) from the single *Igk* allele line. **e**, Diagram of the single Jκ5 allele *v-Abl* line. **f**–**h**, Vκ usage (**f**) and RAG off-target profiles (**g**,**h**) in the single Jκ5 allele line presented as in **b**–**d**. **i**, Diagram of the single Jκ5-Vκ inv *v-Abl* line. **j**–**l**, Vκ usage (**j**) and RAG off-target profiles (**k**,**l**) in the single Jκ5-Vκ inv line presented as in **b**–**d**. Vκ1-135 is over-utilized (**j**), probably owing to its associated transcription. In Fig. 1e,f, Vκ2-137 is equally used, probably owing to its replacement of primary Vκ1-135 inversional rearrangements via deletional secondary rearrangements. Vκ usage data and RAG off-target junctions in the inverted locus are shown in inverted orientation (**j**,**k**). **m**, Chromosome conformation capture (3C)-HTGTS profiles in the *Igk* locus from RAG-deficient *v-Abl* cells baiting from iEκ (red), *Sis* CBE2 (green) and *Cer* CBE1 (blue) and from RAG-deficient primary pre-B cells baiting from *Cer* CBE1 (pink). Asterisks indicate the location of baits. Locations of *Cer*-baited interaction peaks in the Vκ locus significantly above background are indicated with black lines, CBEs in the *Igk* locus are indicated with red (rightward) and blue (leftward) lines. Details on peak calling are provided in  Methods. Vκ utilization and cryptic RSS data are presented as mean ± s.e.m. from 4 (**b**,**d**), 7 (**f**,**h**) or 3 (**j**,**l**) biological repeats; 3C-HTGTS data are presented as mean value from 2 biological repeats. Source Data

To facilitate the assessment of effects of *cis*-acting *Igk* modifications in *v-Abl* cells, we generated a *v-Abl* cell line containing a single *Igk* locus (the single *Igk* allele *v-Abl* line). This line undergoes Vκ-to-Jκ joining nearly identically to its parental line (Fig. 2a,b; compare with Fig. 1b). Long-range RAG chromatin scanning of both the *Igh* and other multi-megabase domains genome-wide can be revealed by highly sensitive HTGTS-V(D)J-seq-based RAG-scanning assays for very low-level RAG-initiated joins between a RC-based bona fide RSS and weak cryptic RSSs as simple as the CAC of the heptamer when in convergent orientation^2,4,5,7^. This assay reveals chromatin regions scanned by RC-based RAG, directionality of exploration, and effects of local chromatin structure on loop extrusion-mediated scanning activity^2,4,5,7^. We used this assay with a Jκ5 bait, which should primarily detect chromosomal joins^8^, to assess RAG scanning versus normal Vκ-to-Jκ joining activity in the single *Igk* allele *v-Abl* line. The results were markedly different from linear strand-specific scanning tracks observed during V_H_-to-DJ_H_ rearrangement^6,7^; indeed, scanning tracks appeared across the Vκ locus on both DNA strands and lacked clear directionality (Fig. 2c,d). These scanning patterns suggested that inversional rearrangements displace *Cer* and *Sis* impediments and place groups of downstream inversional Vκ segments in deletional-orientation upstream of remaining Jκ segments for secondary rearrangements^9,10^, potentially mediated by linear RAG scanning.

**Extended Data Fig. 1:**
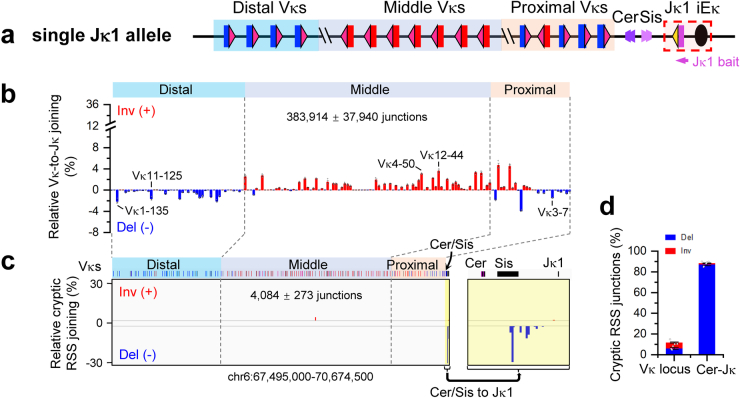
RAG scanning for primary Vκ-to-Jκ1 rearrangement is terminated within *Sis*. Related to Fig. 2. **a**, Diagram of single Jκ1 allele *v-Abl* line. **b**, Relative utilization percentage of individual Vκs in single Jκ1 allele line with Jκ1 bait. **c**, Percentage of pooled RAG off-target junctions in *Igκ* locus from single Jκ1 allele line. Right panel: zoom-in to the region between *Cer* and Jκ, highlighted in yellow. **d**, Percentage of inversional (red) and deletional (blue) cryptic RSS junctions within indicated Vκ locus (chr6:67,495,000-70,657,000) and *Cer*-Jκ regions (chr6:70,657,000- 70,674,500) from single Jκ1 allele line. Vκ utilization and cryptic RSS data are presented as mean ± s.e.m. from 3 biological repeats. Overall figure presentation is as described in Fig. 2. Note that the total on-target and off-target Jκ1 junctions recovered are, respectively, 5-fold and 8-fold greater than those recovered with a Jκ5 bait, consistent with the greater strength of the Jκ1-RSS^47^. Source Data

To more rigorously test the origin of the complex wild-type *v-Abl Igk* scanning patterns, we deleted both Jκ1-4 and the downstream *Igk*-RSS-based deleting element^22^ from the single *Igk* allele *v-Abl* cells, leaving Jκ5 in its normal position relative to iEκ. This ‘single Jκ5 allele’ *v-Abl* line undergoes only primary Vκ-to-Jκ5 rearrangements (Fig. 2e), with rearrangements and scanning patterns representing those that happen during primary Vκ-to-Jκ recombination. Primary bona fide Jκ5 joins to deletional and inversional Vκ segments across the locus were chromosomally retained with patterns somewhat different from those of the parental single *Igk* allele *v-Abl* cells (Fig. 2f; compare with Fig. 2b), probably owing in large part to elimination of secondary rearrangements (see Fig. 2 caption). However, overall findings were clear—primary RAG scanning from the Jκ5-based RC was terminated 8 kb upstream within *Sis* (Fig. 2g,h), despite primary Vκ-to-Jκ joins in the same cells occurring across the locus (Fig. 2f). We also inverted the Vκ locus in the single Jκ5 allele *v-Abl* line to form the ‘single Jκ5–Vκ inv’ line (Fig. 2i). In the single Jκ5–Vκ inv *v-Abl* line, Vκ-to-Jκ rearrangements occurred across the locus, albeit with dominant utilization of the normally distal Vκ1-135 in a proximal position (Fig. 2j); however, primary RAG scanning was still terminated within *Sis* (Fig. 2k,l). Finally, we generated a single Jκ1 allele *v-Abl* line and found that Vκ segments were utilized across the locus (Extended Data Fig. 1a,b); but primary RAG scanning was also terminated within *Sis* (Extended Data Fig. 1c,d).

## Primary Vκ-to-Jκ joining uses short-range diffusion

**Extended Data Fig. 2:**
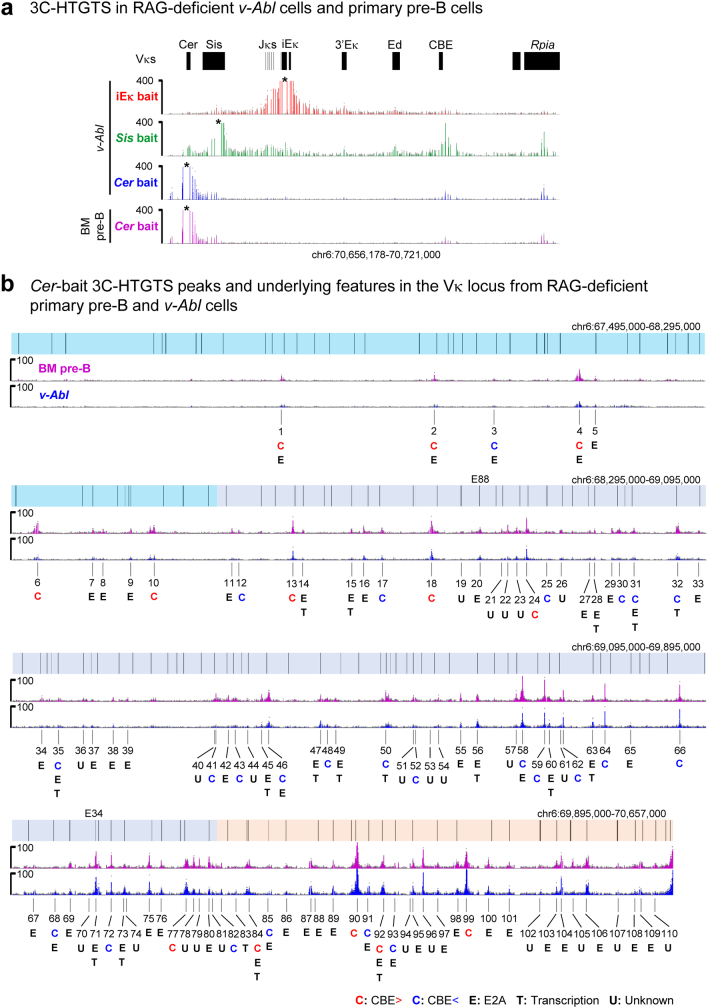
*Cer* interacts with various loop extrusion impediments across the Vκ locus but not substantially with sequences downstream of *Sis*. Related to Fig. 2. **a**, 3C-HTGTS profiles in the *Igκ* downstream region from *Cer* to the downstream *Rpia* gene from RAG-deficient *v-Abl* cells, baiting from iEκ (red), *Sis* CBE2 (green) and *Cer* CBE1 (blue) and from RAG-deficient primary pre-B cells baiting from *Cer* CBE1 (pink). Black asterisks indicate the location of baits. **b**, 3C-HTGTS interaction profiles across the Vκ locus when baiting from *Cer* CBE1 in RAG-deficient primary pre-B (pink) and *v-Abl* (blue) cells. *Cer* interaction peaks and their underlying features in the Vκ locus are shown. A total of 110 peaks were called to be significantly above background in either primary pre-B cells or *v-Abl* cells. Peaks are indicated with black lines and numbered according to their locations from distal to proximal. For each peak, underlying features within ± 1 kb are indicated, including rightward CBE (“C” in red), leftward CBE (“C” in blue), E2A-binding sequence (“E”) and transcription (“T”). Peaks without any obvious underlying features are labeled as unknown (“U”). CBE annotation and transcription were determined based on published CTCF ChIP-seq and GRO-seq data in RAG-deficient *v-Abl* cells^6^. E2A binding was determined based on published E2A ChIP-seq data in RAG-deficient primary pro-B cells^46^. See Methods for more details. 3C-HTGTS data are presented as mean value from 2 biological repeats.

**Extended Data Fig. 3:**
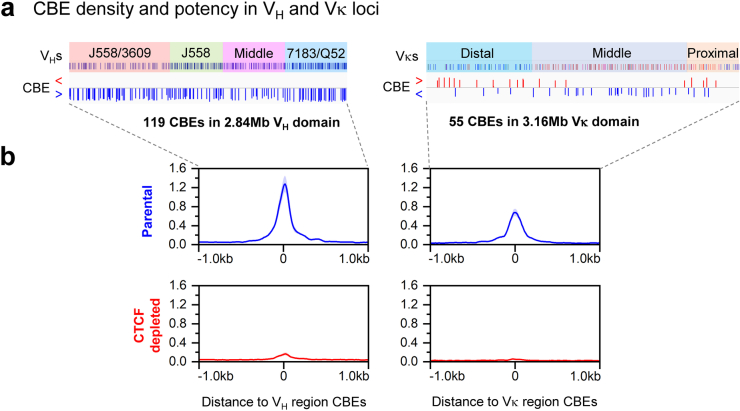
V_H_ locus CBEs are more dense and more potent than Vκ locus CBEs. Related to Fig. 2. **a**, Locations of CBEs in the V_H_ locus (left) and Vκ locus (right). There are 119 annotated CBEs in the 2.84 Mb V_H_ locus and 55 annotated CBEs in the 3.16 Mb Vκ locus. The two loci are shown on the same genomic scale to reflect the difference in CBE density. In the Vκ locus, rightward CBEs are shown in red, leftward CBEs are in blue. In the V_H_ locus, leftward CBEs are shown in red, rightward CBEs are in blue. **b**, Average enrichment of CTCF ChIP-seq signal within ± 1 kb region across all annotated CBEs in the V_H_ locus (left) and Vκ locus (right) in RAG-deficient parental *v-Abl* cells (blue) or CTCF-depleted *v-Abl* cells (red). Data are presented as average signal counts (solid blue or red line) ± s.e.m. (blue or red shade) from 3 biological repeats. The CTCF ChIP-seq data shown were extracted from data deposited in the context of a prior study of RAG-deficient parental and CTCF-depleted *v-Abl* cells^6^. Source Data

Given our findings that RAG does not linearly scan upstream chromatin beyond *Sis* during primary Vκ-to-Jκ rearrangement, we used high-resolution 3C-HTGTS^3^ to explore interactions of the *Igk*-RC, *Sis* or *Cer* with the Vκ locus in RAG-deficient *v-Abl* cells. These analyses revealed that, compared with *Cer*, the *Igk*-RC and *Sis* had little interaction with sequences upstream of *Cer* (Fig. 2m, top 3 tracks). By contrast, *Cer* interacted with more than 100 sites across the Vκ locus in RAG-deficient pre-B cells, many of which were also found in RAG-deficient *v-Abl* lines (Fig. 2m, bottom two tracks). Moreover, *Cer* did not interact substantially with *Igk* sequences, including the RC, downstream of *Sis* (Extended Data Fig. 2a). The strongest *Cer* interactions frequently corresponded to CBEs^23^, but many others corresponded to E2A sites, often in association with transcribed sequences (Extended Data Fig. 2b and Supplementary Data 1). Notably, two previously described Vκ enhancers were in the latter category; deletion of either enhancer affected utilization of nearby Vκ segments^24,25^. As these deletions were done in wild-type cells, additional effects of the enhancer deletions on primary *Igk* rearrangements might be confounded by secondary rearrangements (see example in Fig. 2 caption). Finally, it is notable that these interactions with the *Cer* bait across the Vκ locus occurred with WAPL levels that abrogate interactions of IGCR1 CBE with upstream V_H_ locus scanning impediments^6,26,27^. In this regard, CBEs in the Vκ locus appear less dense and less potent than those in the V_H_ locus (Extended Data Fig. 3a,b). Thus, loop extrusion may proceed more readily across the Vκ locus with high WAPL levels, as found for other multi-megabase loci without strong extrusion impediments in *v-Abl* cells^4^. Internal convergent CBE-based loops in the Vκ locus have been proposed as a major mechanism for bringing Vκ segments into proximity with *Cer*^23^. Our current findings support a mechanism in which juxtaposition of Vκ segments with the *Cer* anchor is mediated by ongoing loop extrusion. During this process CBEs, E2A sites and transcribed sequences act as dynamic impediments^5^ to extend the time for short-range diffusional interactions of Vκ segments with the *Igk*-RC. Transcription can further increase accessibility of RSSs to RAG^28^.

## *Igk*-specific elements promote diffusional joining

**Fig. 3:**
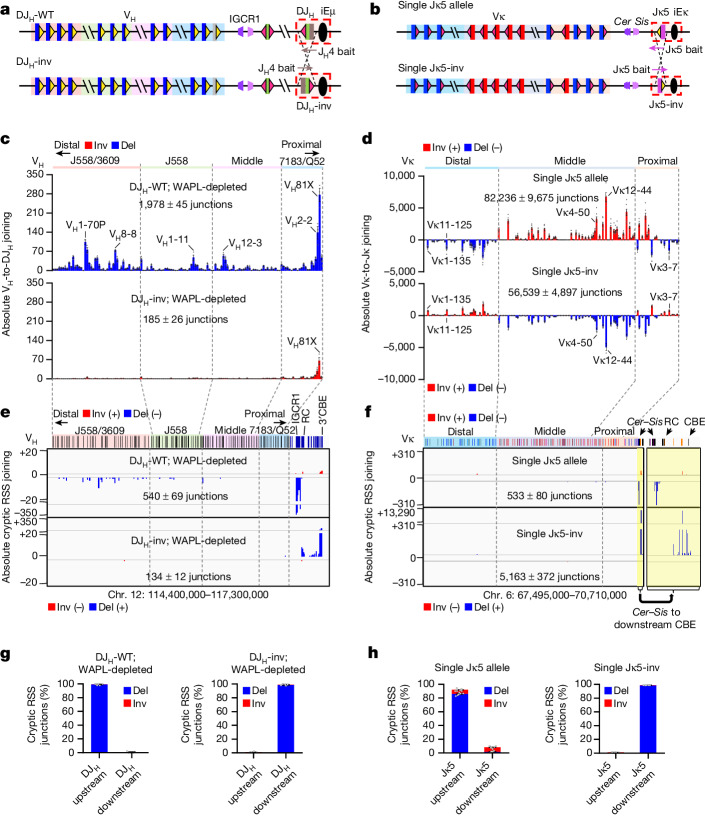
Inverting RC RSS orientation reverses RAG scanning direction and abrogates IgH, but not Igκ, V(D)J recombination. **a**, Diagram of pre-rearranged DQ52J_H_4 in DJ_H_-WT (top) and DJ_H_-inv (bottom) WAPL-degron *v-Abl* lines. **b**, Diagram of Jκ5 in normal (top) and inverted (bottom) orientation from the single Jκ5 and single Jκ5-inv *v-Abl* lines. **c**, Absolute level of individual V_H_ usage from DJ_H_-WT (top) and DJ_H_-inv (bottom) lines with WAPL depletion. **d**, Absolute level of individual Vκ usage from the single Jκ5 allele (top) and single Jκ5-inv (bottom) lines. **e**, Absolute level of pooled RAG off-target junctions from three repeats in the *Igh* locus from DJ_H_-WT (top) and DJ_H_-inv (bottom) lines with WAPL depletion. **f**, Absolute level of pooled RAG off-target junctions from three repeats in the *Igk* locus from the single Jκ5 (top) and single Jκ5-inv (bottom) lines. RAG off-target junction profiles downstream of the *Igk* locus from *Cer* to the downstream CBE are enlarged on the right. The single Jκ5 allele data (**d**,**f**, top) are the same as those shown in Fig. 2f,g; but are plotted here as absolute levels rather than percentages for better alignment and comparison with results from the single Jκ5-inv line. **g**, Percentage of inversional (red) and deletional (blue) cryptic RSS junctions within indicated DJ_H_ upstream (chr. 12:114,666,726–117,300,000) and downstream (chr. 12:114,400,000–114,666,725) region from the DJ_H_-WT (left) and DJ_H_-inv (right) lines with WAPL depletion. **h**, Percentage of inversional (red) and deletional (blue) cryptic RSS junctions within indicated Jκ5 upstream (chr. 6:67,495,000–70,674,000) and downstream (chr. 6: 70,674,001–70,710,000) region from the single Jκ5 allele (left) and single Jκ5-inv (right) lines. Data are presented as mean ± s.e.m. from 3 (**c**,**g**), 7 (**d**, top, **h**, left) or 4 (**d**, bottom, **h**, right) biological repeats. Source Data

To further explore the basis for the differential V(D)J recombination mechanisms in the *Igh* versus *Igk* loci, we generated pre-rearranged DQ52J_H_4 (DJ_H_-WT) and inverted DQ52J_H_4 (DJ_H_-inv) *v-Abl* lines in which WAPL could be depleted (Fig. 3a). In the DJ_H_-WT *v-Abl* line, WAPL depletion activated V_H_-to-DJ_H_ joining and RAG scanning across the V_H_ locus (Fig. 3c,e, top,  g, left). In the WAPL-depleted DJ_H_-inv *v-Abl* line, V_H_-to-DJ_H_ rearrangement was abrogated and RAG scanning was directed downstream through the *Igh* locus to the 3′ CBE cluster (Fig. 3c,e, bottom,  g, right). This finding is notable, as it has been suggested that inverting the V_H_ locus affects V_H_-to DJ_H_ rearrangement by disrupting convergent V_H_ locus CBE-based structure^17^. Our findings from the DJ_H_ inversion rule out this possibility, as the inversion does not alter any CBEs in the *Igh* locus or elsewhere and leaves the RC DJ_H_ inverted in its normal location. Rather, the DJ_H_ inversion only affects the direction of RAG chromatin scanning from the RC. For comparison, we also inverted Jκ5 in the single Jκ5 allele line to generate the ‘single Jκ5-inv’ line (Fig. 3b). Indeed, the Jκ5 inversion redirected RC-bound RAG to scan *Igk* chromatin downstream of the RC to the 3′ *Igk* CBE (Fig. 3f,h). However, other than reversing the orientation by which different Vκ segments joined to the Jκ5, there was little effect on the utilization of upstream Vκ segments across the locus (Fig. 3d). In this regard, as cryptic RSS-based scanning reflects cohesin-mediated loop extrusion past the RC, rather than movement of the RC itself, the inverted Jκ5 would not alter the position of Jκ5-RC-bound RAG relative to *Sis* for short-range diffusional capture of bona fide Vκ-RSSs extruded past *Cer*. These findings from Jκ inversion strongly support the short-range diffusion model for Vκ access to the Jκ-RC and suggest that the *Igk* locus, but not the *Igh* locus, has elements that promote this process.

## Hybrid loci reveal *Igk*-specific elements

**Fig. 4:**
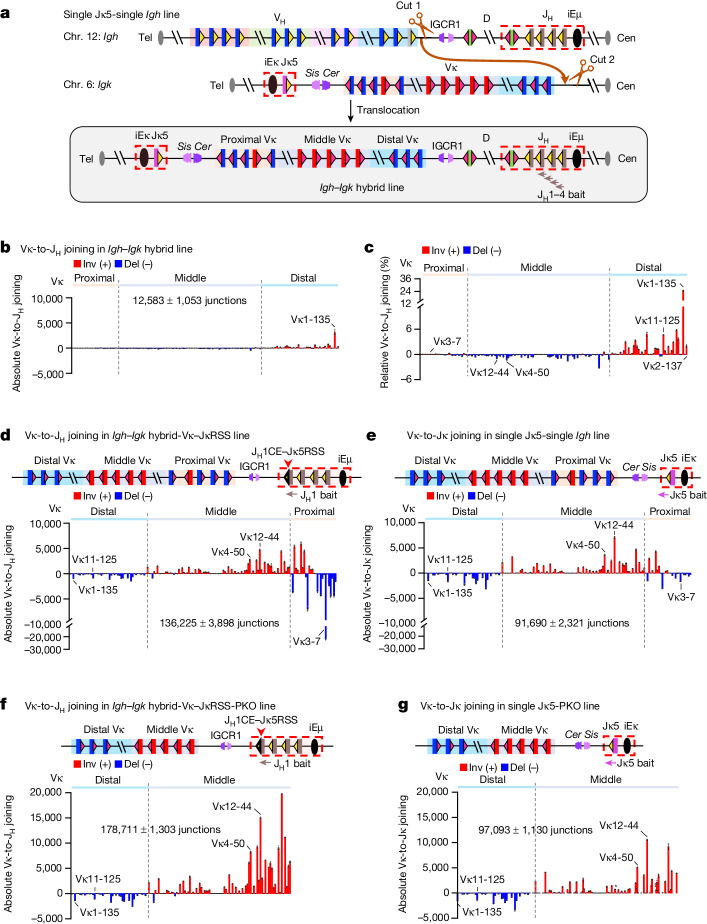
RSS replacements in *Igh–Igk* hybrid loci demonstrate superior strength of *Igk*-RSSs versus *Igh*-RSSs. **a**, Strategy for generating a targeted chromosomal translocation between chr. 12 and chr. 6 in the single Jκ5-single *Igh v-Abl* line. Cut 1 and Cut 2 show the locations of two single guide RNAs (sgRNAs) used for targeting. **b**,**c**, Absolute level (**b**) and relative percentage (**c**) of individual Vκ-to-J_H_ joins in the *Igh–Igk* hybrid line with J_H_1-4 bait. The patterns of Vκ usage in **c** and Fig. 2j are highly similar (two-sided Pearson’s *r* = 0.98, *P* = 9.6 × 10^−110^). **d**, Absolute level of individual Vκ-to-J_H_ joins in the *Igh–Igk* hybrid-Vκ-JκRSS line in which J_H_1-23RSS was replaced with a Jκ5-23RSS, assayed with J_H_1 bait. The patterns of Vκ usage in **d** and Extended Data Fig. 5d are highly similar (two-sided Pearson’s *r* = 0.89, *P* = 1.6 × 10^−56^), but total rearrangement level in **d** is 17-fold higher than that in Extended Data Fig. 5c (*P* = 0.0007; unpaired, two-sided Welch’s *t*-test). Note that Vκ3-7 is highly over-utilized, perhaps promoted by its closely associated E2A site (Supplementary Data 1). **e**, Absolute level of individual Vκ-to-Jκ joins in the single Jκ5-single *Igh* line with Jκ5 bait. **f**, Absolute level of individual Vκ-to-J_H_ joins in the *Igh–Igk* hybrid-Vκ-JκRSS-PKO line in which proximal Vκ domain was deleted, assayed with J_H_1 bait. **g**, Absolute level of individual Vκ-to-Jκ joins in the single Jκ5-PKO line with Jκ5 bait. The patterns of Vκ usage in **f**,**g** are highly similar (two-sided Pearson’s *r* = 0.90, *P* = 1.2 × 10^−49^). Vκ utilization data are presented as mean ± s.e.m. from 3 biological repeats. Source Data

**Extended Data Fig. 4:**
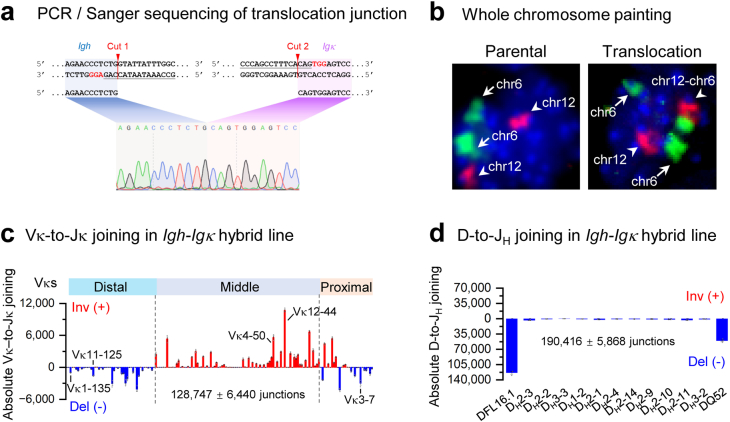
*Igh*-*Igκ* hybrid line generated by targeted chromosomal translocation maintains normal D-to-J_H_ and Vκ-to-Jκ rearrangements. Related to Fig. 4. **a**, Confirmation of translocation junction in *Igh*-*Igκ* hybrid *v-Abl* line (shown in Fig. 4a) by PCR/Sanger sequencing. The sgRNA sequences are underlined, sgRNA cut sites are indicated by red arrows, and the Cas9 PAM sequences are labeled in red. **b**, Whole chromosome painting results with probes tiling chromosome 6 (green) and chromosome 12 (red) in single Jκ5-single *Igh v-Abl* cells (“Parental”, left) and *Igh-Igκ* hybrid *v-Abl* cells (“Translocation”, right). After translocation, a chr12-chr6 fusion chromosome is detected with half of chr6 appended onto chr12. The reciprocal translocation also placed the small telomeric portion (~7 Mb) of chr12 onto chr6, which is below the detectable size of painting experiments. **c**, Absolute level of individual Vκ-to-Jκ joins in *Igh*-*Igκ* hybrid line with Jκ5 bait. **d**, Absolute level of individual D-to-J_H_ joining in *Igh*-*Igκ* hybrid line with J_H_1-4 bait. Vκ and D usage data are presented as mean ± s.e.m. from 3 biological repeats. Source Data

The next major question was to identify the key elements that enable a diffusion-based RC access mechanism to robustly function in *Igk* and not in *Igh*^2^. To address this question, we performed mix-and-match experiments between portions of the two loci. To facilitate these experiments, we used a CRISPR–Cas9-mediated chromosomal translocation targeting approach to generate an *Igh–Igk* hybrid locus in a single Jκ5 allele *v-Abl* line in which we had already deleted one copy of the entire *Igh* locus (Fig. 4a). In this line (*Igh–Igk* hybrid line), the targeted balanced translocation fused the entire *Igk* at a point just upstream of the distal Vκ segments to the downstream portion of *Igh*, starting 85 kb upstream of IGCR1, on a large der(12;6) fusion chromosome (Fig. 4a and Extended Data Fig. 4a,b). Upon G1 arrest and ectopic RAG expression, the *Igh–Igk* hybrid line underwent Vκ-to-Jκ joining similarly to its parental line (Extended Data Fig. 4c; compare with Fig. 4e), and the retained downstream portion of the *Igh* underwent normal levels and patterns of D-to-J_H_ joining^6,7^ (Extended Data Fig. 4d). Thus, the V(D)J recombination activities of the *Igk*-RC and *Igh*-RC are maintained in the *Igh–Igk* hybrid line. To further test the *Igh–Igk* hybrid line, we used HTGTS-V(D)J-seq to assay for joining of the matched J_H_-23RSSs with Vκ-12RSSs across the Vκ locus fused upstream of IGCR1. Remarkably, the J_H_ segments joined to both inversional- and deletional-oriented Vκ segments across the Vκ locus, which is in inverted orientation with respect to J_H_-RSSs (Fig. 4b,c). Although the level of Vκ-to-J_H_ joining across the *Igh–Igk* hybrid locus was only 14% that of Vκ-to-Jκ joining in the normal *Igk* locus (Fig. 4b; compare with Fig. 2j total junction number), this level is far higher than that of residual V_H_-to-DJ_H_ joining across an inverted V_H_ locus in bone marrow pro-B cells^7^. Notably, this long-range Vκ-to-J_H_ joining occurs in *v-Abl* cells, which have high levels of WAPL that essentially abrogate long-range V_H_-to-DJ_H_ joining beyond low-level joining of the most proximal V_H_ segments^7^. Finally, the pattern of Vκ-to-J_H_ joining across the inverted Vκ locus was quite similar to that of Jκ joining to an inverted Vκ locus, with Vκ1-135 dominating rearrangement (Fig. 4c; compare with Fig. 2j).

**Extended Data Fig. 5:**
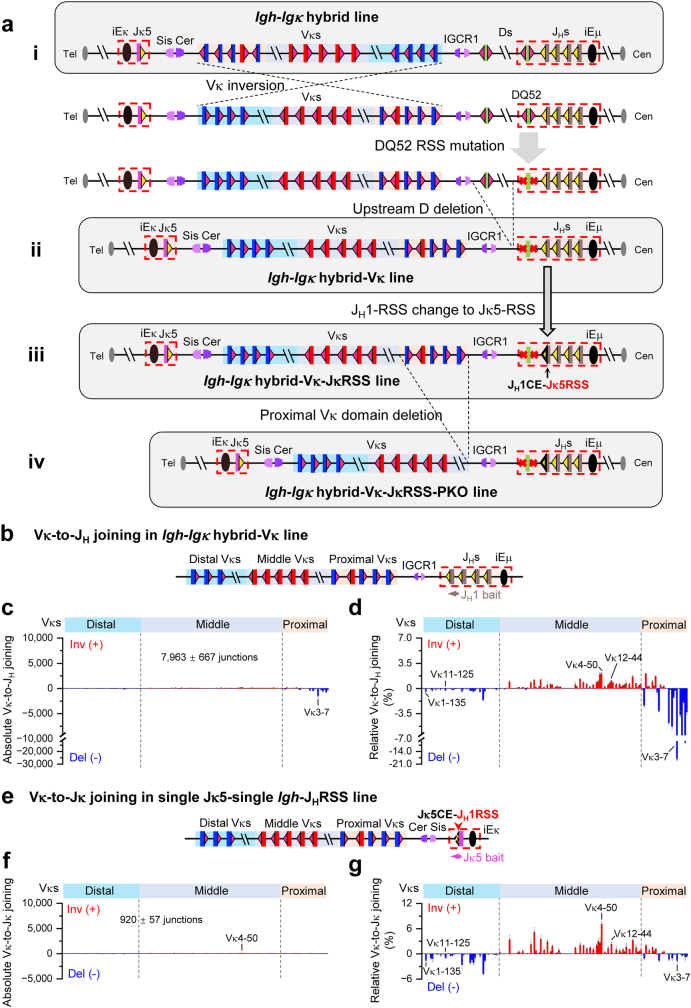
Genetic modifications in the *Igh*-*Igκ* hybrid line and single Jκ5 allele line. Related to Fig. 4. **a**, Diagram of the strategy for various genetic modifications in the *Igh-Igκ* hybrid *v-Abl* line. In brief: (**i**) Diagram of the *Igh-Igκ* hybrid line. (**ii**) Diagram of the *Igh-Igκ* hybrid-Vκ line which was generated from the *Igh-Igκ* hybrid line by inverting the whole Vκ locus, mutating both RSSs of DQ52, and deleting all upstream Ds, as illustrated in the diagrams just above. (**iii**) Diagram of the *Igh-Igκ* hybrid-Vκ-JκRSS line which was generated from the *Igh-Igκ* hybrid-Vκ line by replacing the J_H_1-23RSS with a Jκ5-23RSS. (**iv**) Diagram of the *Igh-Igκ* hybrid-Vκ-JκRSS-PKO line which was generated from the *Igh-Igκ* hybrid-Vκ-JκRSS line by deleting the proximal Vκ domain. See Methods for more details. **b**, Diagram of *Igh*-*Igκ* hybrid-Vκ line, as shown in **a**(**ii**). **c**, Absolute level, and **d**, relative percentage of individual Vκ-to-J_H_ joins in *Igh*-*Igκ* hybrid-Vκ line with J_H_1 bait. The patterns of distal and middle Vκ usage in the *Igh-Igκ* hybrid-Vκ line (**d**) and the single Jκ5-single *Igh* line (Fig. 4e) are similar (Two-sided Pearson’s *r* = 0.70, *P* = 2.2e-21). **e**, Illustration of single Jκ5-single *Igh*-J_H_RSS line, in which Jκ5-23RSS was replaced with J_H_1-23RSS. **f**, Absolute level, and **g**, relative percentage of individual Vκ-to-Jκ joins in single Jκ5-single *Igh*-J_H_RSS line with Jκ5 bait. Total rearrangement level in **f** is 100-fold lower than that in Fig. 4e (*P* = 0.0006; unpaired, two-sided Welch *t*-test). Vκ usage data are presented as mean ± s.e.m. from 3 biological repeats. Source Data

**Extended Data Fig. 6:**
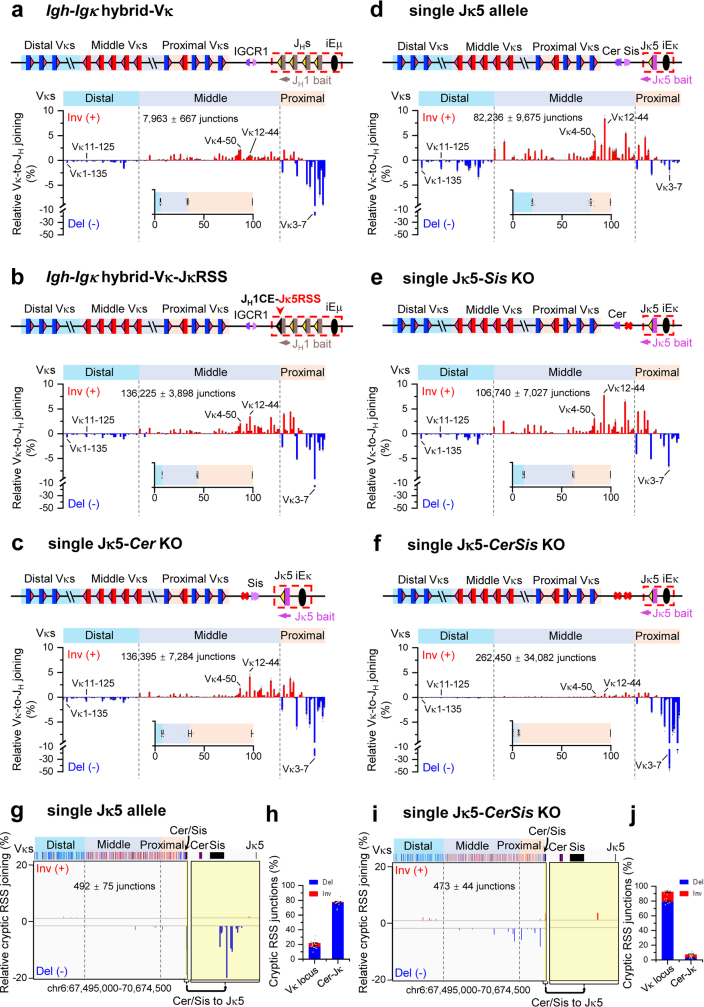
IGCR1 is a weaker anchor than *Cer-Sis* in preventing over-utilization of proximal deletional Vκs. Related to Fig. 4. **a**-**f**, Relative utilization percentage of individual Vκs in *Igh*-*Igκ* hybrid-Vκ line (**a**), *Igh-Igκ* hybrid-Vκ-JκRSS line (**b**), single Jκ5-*Cer* KO line (**c**), single Jκ5 allele line (**d**), single Jκ5-*Sis* KO line (**e**), single Jκ5-*CerSis* KO line (**f**) analyzed with indicated baits. Bar graph in the inset of each panel shows the percentage of distal (blue), middle (gray) and proximal (orange) Vκ domain usage from the corresponding line. **g**, Percentage of pooled RAG off-target junctions in *Igκ* locus from single Jκ5 allele line. Right panel: zoom-in to the region between *Cer* and Jκ, highlighted in yellow. **h**, Percentage of inversional (red) and deletional (blue) cryptic RSS junctions from single Jκ5 allele line within indicated regions as in Fig. 2d. **i**-**j**, RAG off-target profiles in single Jκ5-*CerSis* KO line presented as in **g**-**h**. A group of aberrant pseudo-normal coding-end junctions^48^ to sequences near the *Igκ* downstream CBE were excluded^4^. The patterns of Vκ usage in **a**, **b** and **c** are highly similar (**a** and **c**, Two-sided Pearson’s *r* = 0.91, *P* = 1.5e-63; **b** and **c**, Two-sided Pearson’s *r* = 0.97, *P* = 1.0e-99). The data shown in **a** and **d** are the same as that shown in Extended Data Fig. 5d and Fig. 2f, the data shown in **g** and **h** are the same as that shown in Fig. 2g and h, respectively, plotted here for better alignment and comparison with other results. Vκ utilization and cryptic RSS data are presented as mean ± s.e.m. from 3 (**a**,**b**,**e**), 4 (**c**), 7 (**d**,**h**) or 6 (**f**,**j**) biological repeats. Source Data

**Extended Data Fig. 7:**
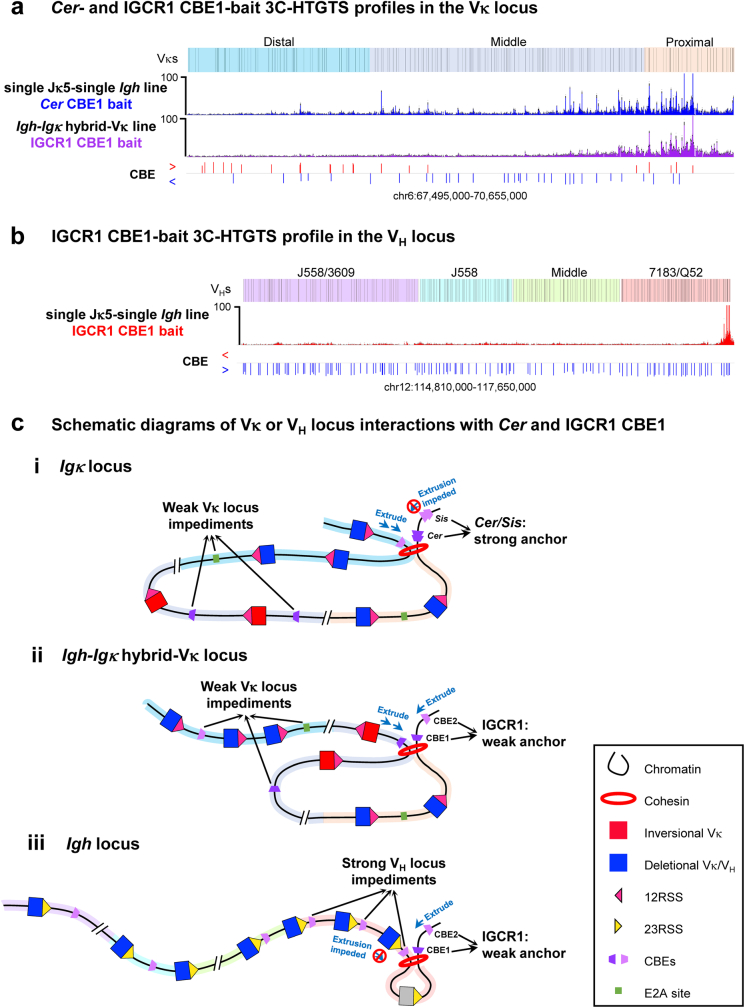
Interactions of IGCR1 or *Cer-Sis* with V_H_ or Vκ locus in *v-Abl* cells. Related to Fig. 4 and Extended Data Fig. 3. **a**, **Upper panel**: 3C-HTGTS profiles in the Vκ locus from single Jκ5-single *Igh v-Abl* line baiting from *Cer* CBE1. **Lower panel**: 3C-HTGTS profiles in the Vκ locus from *Igh*-*Igκ* hybrid-Vκ *v-Abl* line baiting from IGCR1 CBE1. **b**, 3C-HTGTS profiles in the V_H_ locus from single Jκ5-single *Igh* line baiting from IGCR1 CBE1. CBE sites are shown in **a** and **b** with orientations labeled as in Extended Data Fig. 3a. 3C-HTGTS data are presented as mean ± s.e.m. from 3 biological repeats (**a**) or as mean value from 2 biological repeats (**b**). **c**, Schematic loop domain illustrations of *Igκ*, *Igh*, and *Igh-Igκ* hybrid-Vκ loci based on 3C-HTGTS data shown in **a** and **b**. (**i**) In *Igκ* locus, the strong anchoring activity of *Cer-Sis*, coupled with relatively weak impediments in the Vκ locus, allows loop extrusion anchored at *Cer* to extend across the distal, middle and proximal Vκ domains, as shown in **a**, **upper panel**. (**ii**) In *Igh-Igκ* hybrid-Vκ locus, loop extrusion anchored at IGCR1 can extend a considerable distance into proximal and middle Vκ domains with weak Vκ locus impediments, but does not extend as far as that in (**i**), because IGCR1 is a less stable anchor than *Cer-Sis* and more likely to be disassembled before loop extrusion has a chance to proceed into the distal Vκ locus, as shown in **a**, **lower panel**. (**iii**) In *Igh* locus without WAPL down-regulation, strong V_H_ locus impediments only allow loop extrusion to bring the most proximal V_H_ region to IGCR1, while upstream interactions are completely blocked by the “wall” of proximal V_H_ CBEs, as shown in **b**. Elements and proteins illustrated are indicated in the box. Source Data

For further comparison of Vκ-to-J_H_ rearrangement patterns and levels, we used a CRISPR–Cas9 approach to modify the *Igh–Igk* hybrid locus by first inverting the Vκ locus, so that it is in the same relative orientation to J_H_-RSSs as the normal Vκ locus is to Jκ-RSSs (Extended Data Fig. 5a). To avoid potential confounding effects of competing D-to-J_H_ rearrangements, we deleted all D segments upstream of DQ52 and inactivated both DQ52 RSSs by targeted mutation (Extended Data Fig. 5a), leaving inactivated DQ52 in its normal position to retain its germline promoter and transcription to contribute to *Igh-*RC activity^29^. This further modified *v-Abl* line was termed the ‘*Igh–Igk* hybrid-Vκ line’ (Extended Data Fig. 5a). HTGTS-V(D)J-seq analyses of Vκ-to-J_H_ joining in the *Igh–Igk* hybrid-Vκ line revealed J_H_ joining to both deletional- and inversional-oriented Vκ segments across the locus, but at approximately 9% the level of bona fide Vκ-to-Jκ5 joins (Extended Data Fig. 5b–d; compare with Fig. 4e). Whereas the joining patterns of middle and distal Vκ segments were very similar to those of the normal locus, relative utilization of the proximal deletional-oriented Vκ segments was increased (Extended Data Fig. 5d; compare with Fig. 4e). The increased proximal Vκ utilization phenotype could potentially reflect leakiness of the IGCR1 scanning impediment, enabling low-level RAG linear scanning to pass into the proximal Vκ locus versus the *Igh* locus in which IGCR1 is backed up by proximal V_H_-associated CBE impediments^16,26^. To test this possibility, we compared Vκ rearrangement patterns of the *Igh–Igk* hybrid-Vκ line to those of single Jκ5 lines in which either *Cer*, *Sis* or both *Cer* and *Sis* were deleted (Extended Data Fig. 6). Consistent with prior analyses^12,30^, *Cer* alone maintained nearly wild-type joining patterns, whereas the absence of both *Cer* and *Sis* greatly increased proximal Vκ rearrangements at the expense of distal Vκ rearrangements (Extended Data Fig. 6e,f). *Cer* and *Sis* deletion also led to extended linear RAG scanning from the ectopic primary *Igk*-RC into the proximal Vκ region (Extended Data Fig. 6g–j). Notably, the rearrangement patterns in cells with *Sis* alone in which *Cer* was deleted were remarkably similar to those of the *Igh–Igk* hybrid-Vκ line (compare Extended Data Fig. 6a,c). Together, these results support the notion that relative leakiness of the IGCR1 CBE-based impediment, as compared to *Cer*–*Sis* deletion, results in increased utilization of proximal Vκ segments in the *Igh–Igk* hybrid-Vκ line. Finally, 3C-HTGTS analyses of the hybrid locus confirmed both the greater strength of the *Cer–Sis* anchor compared with IGCR1 and the relative weakness of Vκ locus loop extrusion impediments compared with those of the V_H_ locus (Extended Data Fig. 7).

As nearly all Vκ segments show low-level rearrangement to J_H_ segments in the presence of IGCR1, a candidate element that could enhance diffusional capture by the *Igh*-RC would be the Vκ-associated RSSs; which could, in theory, mediate this activity by being stronger than V_H_-RSSs. In this regard, proximal V_H_ RSSs appear very weak in promoting V_H_-to-DJ_H_ joining in the absence of directly associated CBEs that increase their interaction with the *Igh*-RC^3^. This model leads to the further hypothesis that a potential limiting factor for the overall level of Vκ-to-J_H_ joins versus Vκ-to-Jκ joins, is relative strength of the Jκ-RSSs versus J_H_-RSSs. To test this possibility, we further modified the *Igh–Igk* hybrid-Vκ locus by replacing J_H_1-23RSS with Jκ5-23RSS to generate the ‘*Igh–Igk* hybrid-Vκ-JκRSS’ line (Extended Data Fig. 5a), in which the entire downstream *Igh* locus including IGCR1, the *Igh*-RC and downstream sequences were in the same position as in the *Igh–Igk* hybrid-Vκ line. Remarkably, the pattern of Vκ-to-J_H_ rearrangements in the *Igh–Igk* hybrid-Vκ-JκRSS line was very similar to that of the parental *Igh–Igk* hybrid-Vκ line (Fig. 4d; compare with Extended Data Fig. 5d), but the absolute level of rearrangements to Vκ segments across the locus increased approximately 17-fold (compare Fig. 4d with Extended Data Fig. 5c) to levels slightly higher than those of Vκ-to-Jκ joining in the single Jκ5-single *Igh* line (Fig. 4e). To eliminate the dominance of Vκ3-7 (Fig. 4 caption) and, to a lesser extent, other proximal Vκ segments associated with leaky direct scanning through IGCR1 in the *Igh–Igk* hybrid-Vκ-JκRSS line, we deleted the most proximal deletional and inversional Vκ segments from this line to generate the ‘*Igh–Igk* hybrid-Vκ-JκRSS-PKO’ line (Extended Data Fig. 5a). Of note, the pattern of Vκ-to-J_H_ rearrangements in the *Igh–Igk* hybrid-Vκ-JκRSS-PKO line was very similar to that in the single Jκ5 line with the same proximal Vκ deletion (single Jκ5-PKO line; Fig. 4f,g), with the absolute level of Vκ rearrangements across the *Igh–Igk* hybrid-Vκ-JκRSS-PKO locus approximately twofold higher than that of the single Jκ5-PKO line (Fig. 4f,g). Finally, to further test the relative RSS strength model, we performed the reciprocal experiment of replacing the Jκ5-23RSS with a J_H_1-23RSS in the single Jκ5 allele *v-Abl* line (Extended Data Fig. 5e). Indeed, the J_H_1-RSS supported only low-level Vκ-to-Jκ joining (1% the level supported by the Jκ5-RSS) (Extended Data Fig. 5f; compare with Fig. 4e), but essentially all Vκ segments were utilized (Extended Data Fig. 5g). The findings from our hybrid locus experiments demonstrate that strong *Igk*-RSSs are the major determinant of why *Igk*, but not *Igh*, supports robust diffusion-mediated V(D)J recombination.

## *Igk*-RSSs are much stronger than *Igh*-RSSs

**Fig. 5:**
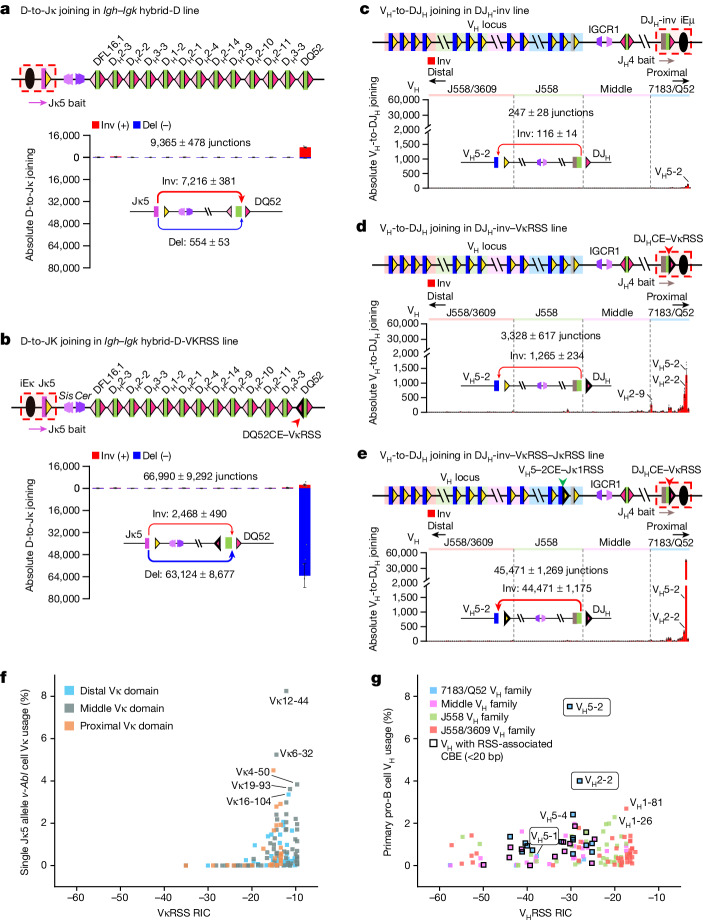
*Igk*-RSSs enhance diffusional D-to-Jκ joining in the *Igh–Igk* hybrid locus and activate inversional V_H_-to-DJ_H_ joining in the *Igh* locus. **a**,**b**, Absolute level of individual D-to-Jκ joins in the *Igh–Igk* hybrid-D line (**a**) and the *Igh–Igk* hybrid-D-VκRSS line in which the DQ52 upstream 12RSS was replaced with a Vκ12-44 12RSS (**b**), assayed with Jκ5 bait. Deletional DQ52-to-Jκ5 joining in **b** is 114-fold higher than that in **a** (*P* = 0.0008). **c**–**e**, Absolute level of individual inversional V_H_-to-DJ_H_ joins in the DJ_H_-inv line (**c**), the DJ_H_-inv-VκRSS line, in which DQ52 upstream 12RSS was replaced with a Vκ11-125 12RSS (**d**) and the DJ_H_-inv-VκRSS-JκRSS line, in which V_H_5-2 23RSS was replaced with a Jκ1-23RSS (**e**), assayed with J_H_4 bait. Total rearrangement level in **d** is 13-fold higher than that in **c** (*P* = 0.0153). Inversional V_H_5-2 usage level in **e** is 35-fold higher than that in **d** (*P* = 0.0005) and 383-fold higher than that in **c** (*P* = 0.0007). In **a**–**e**, red arrows show inversional joins and blue arrows show deletional joins. Corresponding junction numbers are shown. Arrow thickness represents relative amounts of junctions. **f**, Comparison of relative Vκ usage in the single Jκ5 allele *v-Abl* cells with Vκ-RSS RIC scores calculated using the Recombination Signal Sequences Site^33^ (http://www.itb.cnr.it/rss). Vκ segments are colour-coded according to the three Vκ domains with names indicated for highly used Vκ segments. **g**, Comparison of relative V_H_ usage in primary pro-B cells^7^ with V_H_-RSS RIC scores. V_H_ segments are colour-coded according to the four V_H_ domains, and square black outlines indicate V_H_ segments with CBEs within 20 bp of their RSSs. The circled V_H_5-1, V_H_5-2 and V_H_2-2 have been shown to depend on associated CBEs for robust utilization^3^. D and V_H_ utilization data are presented as mean ± s.e.m. from 4 (**a**,**d**), 6 (**b**) and 3 (**c**,**e**) biological repeats. *P* values were calculated with unpaired, two-sided Welch’s *t*-test. Source Data

**Extended Data Fig. 8:**
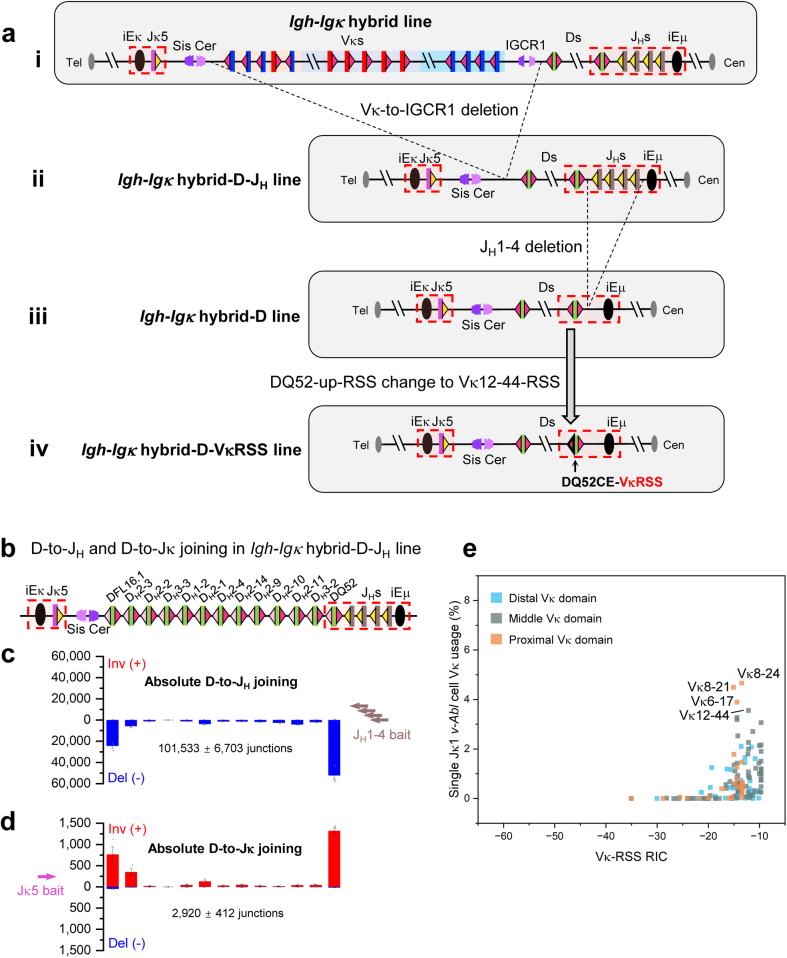
Genetic modifications in the *Igh-Igκ* hybrid-D line, and correlation of Vκ usage with RIC score. Related to Fig. 5. **a**, Diagram of the strategy for various genetic modifications in the *Igh-Igκ* hybrid-D *v-Abl* line. (**i**) Diagram of the *Igh-Igκ* hybrid line. (**ii**) Diagram of the *Igh-Igκ* hybrid-D-J_H_ line which was generated from the *Igh-Igκ* hybrid line by deleting all Vκs and IGCR1. (**iii**) Diagram of the *Igh-Igκ* hybrid-D line which was generated from the *Igh-Igκ* hybrid-D-J_H_ line by deleting J_H_1-4. (**iv**) Diagram of the *Igh-Igκ* hybrid-D-VκRSS line which was generated from the *Igh-Igκ* hybrid-D line by replacing the DQ52 upstream 12RSS with a Vκ12-44 12RSS. See Methods for details. **b**, Diagram of the *Igh-Igκ* hybrid-D-J_H_ line as illustrated in **a**(**ii**). **c**, Absolute level of D-to-J_H_ joins baiting from J_H_1-4 in the *Igh-Igκ* hybrid-D-J_H_ line. **d**, Absolute level of D-to-Jκ joins baiting from Jκ5 in the *Igh-Igκ* hybrid-D-J_H_ line. D utilization data are presented as mean ± s.e.m. from 3 biological repeats. **e**, Comparison of relative Vκ usage in single Jκ1 allele *v-Abl* cells with Vκ-RSS RIC scores. Vκs are color-coded according to the three Vκ domains with names indicated for highly used Vκs. Source Data

To directly test relative strength of *Igh* D-12RSSs versus that of a Vκ-12RSS in the context of short-range diffusional joining to the Jκ5-based RC, we used a CRISPR–Cas9-mediated approach to further modify the *Igh–Igk* hybrid locus. Specifically, we generated a deletion from 5,123 bp upstream of *Cer* (just downstream of the Vκ locus) to a point 453 bp upstream of DFL16.1 in the *Igh–Igk* hybrid locus to generate the ‘*Igh–Igk* hybrid-D-J_H_’ line (Extended Data Fig. 8a). In this line, the downstream portion of *Igk* including the Jκ5-based RC and *Cer–Sis* elements were placed just upstream of the DFL16.1, the 12 downstream D segments, and the J_H_-RC (Extended Data Fig. 8a). We first assayed for D-to-J_H_ rearrangements in the *Igh–Igk* hybrid-D-J_H_ line and found the vast majority to be deletional and mostly utilize DFL16.1 and DQ52 (Extended Data Fig. 8b,c), similar to normal deletional-dominated patterns (Extended Data Fig. 4d). We also found D-to-Jκ5 rearrangements at much lower levels; but, nearly all were inversional to DQ52 and DFL16.1 (Extended Data Fig. 8d), consistent with Jκ-RC-bound RAG accessing these D segments by short-range diffusion across *Cer–Sis*, which is dominated by their stronger downstream D-RSSs^5^. Indeed, for D-to-J_H_ joining, the various D downstream RSSs are stronger than their upstream RSSs, with the DQ52 downstream RSS being the strongest^5^. To develop a line for directly comparing relative ability of a Vκ-RSS versus D-RSSs to mediate D-to-Jκ rearrangements, we deleted all J_H_ segments from the *Igh–Igk* hybrid-D-J_H_ line to generate the ‘*Igh–Igk* hybrid-D’ line (Fig. 5a and Extended Data Fig. 8a). Activation of V(D)J recombination in this line resulted in primarily DQ52 joining to Jκ5 in which the strong downstream DQ52-RSS dominated rearrangements that were predominantly (13-fold) inversional versus deletional (Fig. 5a). Again, the high level of inversional DQ52-to-Jκ5 joining is consistent with short-range diffusional access across *Cer–Sis*. Remarkably, replacement of the weaker upstream DQ52-12RSS with the 12RSS of the highly utilized Vκ12-44 in the *Igh–Igk* hybrid-D line led to a 114-fold increase in the level of Jκ5 deletional joining to DQ52 (Fig. 5b; compare with Fig. 5a), a level approximately 26-fold greater than that of inversional joining mediated by the downstream DQ52-RSS (Fig. 5b). These results demonstrate the remarkable functional strength of the Vκ-12RSS, compared with the DQ52 downstream 12RSS and all other *Igh* D-12RSSs in mediating diffusion-based D-to-Jκ5 rearrangements.

## *Igk*-RSSs programme diffusional joining in *Igh*

We tested the relative ability of the frequently utilized Vκ11-125 RSS versus that of the upstream DQ52-RSS to mediate joining of proximal V_H_ segments to the inverted DQ52J_H_4-based RC. For this experiment, we did not deplete WAPL to leave IGCR1 CBE impediments fully functional to enforce short-range diffusion mediated joining of the most proximal V_H_ segments. With high WAPL levels, distal V_H_ segments are prevented from being extruded past IGCR1 by many robust CBE impediments associated with proximal and middle V_H_-RSSs^6,26,27^ (Extended Data Fig. 7b). In the DQ52J_H_4-inverted line, we found very low levels of inversional joining to proximal V_H_5-2 mediated by the inverted upstream DQ52-12RSS (Fig. 5c). However, upon replacement of this DQ52-12RSS with the Vκ11-125-12RSS, inversional rearrangements increased approximately 13-fold, predominantly to V_H_5-2 but at lower levels to additional proximal V_H_ segments (Fig. 5d; compare with Fig. 5c). To test the cooperative ability of *Igk*-RSSs to promote inversional rearrangement, we replaced the V_H_5-2-23RSS with the Jκ1-23RSS in the *v-Abl* line in which the DQ52-12RSS was replaced with the Vκ11-125-12RSS. Remarkably, the Jκ1-RSS replacement led to a further 35-fold increase in V_H_5-2 to inverted DQ52J_H_4 joining (Fig. 5e; compare with Fig. 5d). Indeed, the overall increase in V_H_5-2 to inverted DQ52J_H_4 joining was more than 380-fold (Fig. 5e; compare with Fig. 5c). This joining level approaches that of direct deletional V_H_5-2-to-DFL16.1J_H_4 joining in the absence of IGCR1^3^. Together, these findings demonstrate that paired *Igk* 12 and 23 RSSs programme the *Igh* to undergo robust V_H_-to-DJ_H_ inversional joining mediated by short-range diffusion.

## Relevance of RSS RIC scores to joining mechanism

The theoretical strength of given 12RSSs and 23RSSs, respectively, has been estimated on the basis of an algorithm that assesses recombination information content (RIC) scores of their sequence^31–33^. Previous studies failed to detect strong correlations between RIC scores of V_H_-RSSs or, to a lesser extent, Vκ-RSSs and their utilization frequency^34–37^. Predicted RIC thresholds for 12RSSs and 23RSSs are −38.81 and −58.45, respectively^31,33^, with increasing RIC scores proposed to reflect increasing RSS strength. Because 12RSS and 23RSS RIC scores cannot be directly compared^31,32^, we examined Vκ-12RSS or V_H_-23RSS RIC scores and corresponding Vκ or V_H_ usage in, respectively, single Jκ5 allele *v-Abl* cells to focus on primary Vκ rearrangements, or normal pro-B cells to focus on V_H_ rearrangements in the context of physiological WAPL down-regulation^7^. Most highly used Vκ-12RSSs in single Jκ5 allele *v-Abl* cells have RIC scores tightly clustered between −16 and −8, with −8 being the highest observed (Fig. 5f); Vκ-12RSSs with RICs below −20 are rarely utilized (Fig. 5f). Similar results were observed in single Jκ1 *v-Abl* cells (Extended Data Fig. 8e). Approximately 26% of Vκ-RSSs with high RIC scores are rarely utilized. The reason for this is unknown; but one possibility is that these Vκ segments are not in chromatin regions that promote sufficient accessibility to the RAG-bound RC^36,37^. V_H_-23RSSs, which span a broader range of RIC scores from −57 to −16, support a similar range of utilization levels, with the exception of proximal V_H_5-2 and V_H_2-2 that have lower RIC scores but very high utilization (Fig. 5g). But, robust rearrangement of these two V_H_ segments is promoted by CBEs within 20 bp of their RSSs, which promotes accessibility by enhancing V_H_-RSS contact with the RC during RAG scanning^3^. Indeed, inactivation of these RSS-associated CBEs reduces utilization to near baseline, consistent with RSSs themselves being very weak^3^. Likewise, adding an associated CBE to the barely utilized, low RIC score proximal V_H_5-1 RSS makes it the most highly utilized^3^. Transcriptional impediments are likely to function similarly for more distal V_H_-RSSs^5–7^; although more distal V_H_-RSSs also have higher RIC scores (Fig. 5g). Notably, 28 of the most proximal V_H_ segments have CBEs within 20 bp of their RSSs; but, none of the 103 Vκ segments are associated with such proximal CBEs^37^.

## Discussion

**Extended Data Fig. 9:**
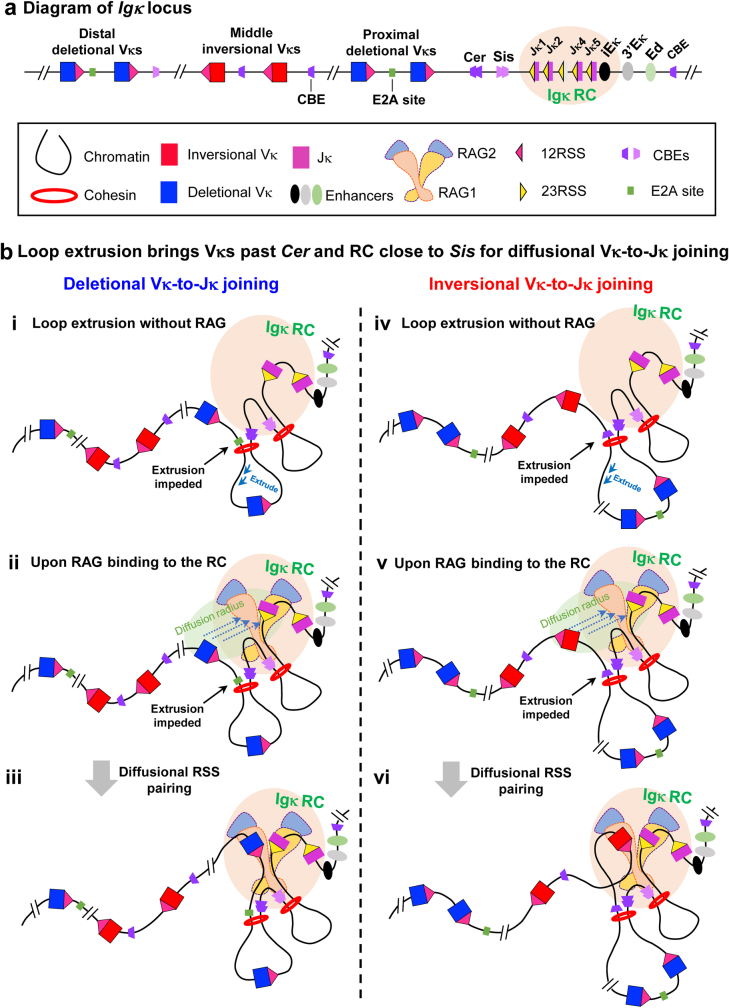
Working model for short-range diffusion-mediated primary *Igκ* V(D)J recombination. **a**, Diagram of *Igκ* (not to scale). Elements and proteins illustrated are indicated in the box. **b**, Working model. (**i**) Loop extrusion of downstream chromatin through a cohesin ring impeded in the upstream direction at *Sis* juxtaposes the RC, a downstream impediment, to *Sis*. Simultaneously, loop extrusion of upstream chromatin through a cohesin ring impeded in the downstream direction at *Cer* brings the Vκ locus past *Cer*. (**ii**) During extrusion past *Cer*, relatively weak extrusion impediments, including CBEs and E2A sites (illustrated) across the Vκ locus dynamically impede extrusion at *Cer*, providing more opportunity for Vκ-RSSs to remain in short-range diffusion distance for interactions with RC-bound RAG. (**iii**) Binding of paired strong Vκ-RSSs to the RAG-1 active site across from strong Jκ-RSSs promote robust cleavage and/or joining. (**iv**-**vi**) Only a fraction of Vκ-RSSs brought into diffusion range pair with Jκ-RSSs, allowing extrusion to continue upstream where impediments slow down extrusion past *Cer*, providing opportunity for additional Vκ-RSSs to interact with RC-bound RAG. These panels diagram use of inversional-oriented Vκs, which can interact by the same short-range diffusion process outlined for deletional Vκs. The diagram is simplified to provide a general overview of the proposed mechanism, for which details await high resolution studies. Due to relatively weak *Igκ* impediments, this model is compatible with cohesin loading across the Vκ locus^2^. Also, RAG is likely not continuously bound to the RC^2,7^, allowing extrusion to continue past *Cer*. These latter features could allow active RAG-bound RCs to initiate the process at different points across the Vκ locus to optimize diverse Vκ utiliation^2,7^. Human *Igκ,* which undergoes deletional and inversional joining, has *Cer-Sis*-like elements in the Vκ-Jκ interval^12,49,50^ and high Vκ-RSS RIC scores^33^, consistent with employing a similar primary rearrangement mechanism to mouse *Igκ*.

The molecular basis by which *Igk*, but not *Igh*, is able to utlilize a diffusion-based mechanism to promote both deletional and inversional joining was a long-standing mystery. Our studies reveal that *Igk* and *Igh* evolved RSSs with distinctly different strength to carry out their distinct mechanisms of long-range V(D)J recombination. Until now, RSSs were not known to function in the broad context of mediating distinct V(D)J recombination mechanisms between loci. Long ago, we found that differential RSS strength mediates ordered Dβ-to-Jβ and Vβ-to DJβ joining by a “beyond 12/23” mechanism^38,39^; and, more recently, weaker Vβ-RSSs were implicated in facilitating allelic exclusion of Vβ-to-DJβ joining^40^. *Igh* DQ52 evolved a relatively strong downstream RSS to enforce deletional joining to closely linked J_H_-RSSs via short-range diffusion; correspondingly, when inverted the strong downstream DQ52-RSS mediates robust inversional joining^5^. Yet, insertion of an inverted DQ52 in an upstream position beyond diffusion range led the weaker upstream DQ52-RSS—now facing downstream—to dominantly generate deletional rearrangements to J_H_ via linear RAG scanning^5^. The relative strength of *Igk*-RSSs is underscored by our finding that a Vκ-12RSS is orders of magnitude stronger than the downstream DQ52-12RSS in mediating diffusional joining in the context of the *Cer–Sis* impediment. Similarly, whereas *Igh* IGCR1 is weaker in impeding RAG scanning than *Cer–Sis*, in the *Igh–Igk* hybrid-Vκ locus, it supports substantial diffusional Vκ capture and joining by RAG bound to a downstream *Igh*-RC in which the J_H_-RSS is replaced with a Jκ-RSS. Moreover, robust diffusional joining of V_H_ to an inverted DJ_H_-RC occurs only when V_H_-RSS and DJ_H_-RSS are replaced with 12/23-matched *Igk*-RSSs. Whereas single Vκ- or Jκ-RSSs increase diffusion-mediated joining in the above contexts, highly robust joining occurs only with 12/23 matched *Igk-*RSSs, either through multiplicative effects and/or by more robust pairing. In summary, our findings indicate that the *Igk* evolved both a robust *Cer* diffusion platform and strong RSSs that function robustly in the context of more transient RC interactions that likely occur during diffusion-mediated primary Vκ-to-Jκ joining (Extended Data Fig. 9). By contrast, weak *Igh*-RSSs and a less robust IGCR1 impediment probably evolved to facilitate mediation of V_H_ utilization by WAPL down-regulated modulation of scanning impediments during long-range linear RAG scanning. Finally, our studies suggest the testable hypothesis that *Igk* secondary rearrangements with *Cer–Sis* deleted or displaced occur by linear RAG scanning.

## Methods

### Experimental procedures

Statistical methods were not used to predetermine sample size. Experiments were not randomized. Investigators were not blinded to allocation during experiments and outcome assessment.

### Mice

Wild-type 129SV mice were purchased from Taconic Biosciences. All mouse work was performed in compliance with all the relevant ethical regulations established by the Institutional Animal Care and Use Committee (IACUC) of Boston Children’s Hospital and under protocols approved by the IACUC of Boston Children’s Hospital. Mice were maintained on a 14-h light/10-h dark schedule in a temperature (22 ± 3 °C) and humidity (35% ~ 70% ± 5%)-controlled environment, with food and water provided ad libitum. Male and female mice were used equally for all experiments.

### Generation and characterization of the entire Vκ locus inversion mouse model

The CRISPR–Cas9-mediated entire Vκ locus inversion modifications were made on one *Igk* allele in the TC1 embryonic stem (ES) cell line. Targeting of the ES cells was performed using sgRNA1 and sgRNA2 as previously described^41^. Positive clones with 3.1 Mb Vκ locus inversion were identified by PCR and confirmed by Sanger sequencing. After testing negative for mycoplasma, the ES clone with Vκ inversion was injected into RAG2-deficient blastocysts to generate chimeras^42^. The chimeric mice were bred with wild-type 129SV mice for germline transmission of the targeted inversion, and bred to homozygosity. Sequences of all sgRNAs and oligonucleotides mentioned in this section and sections below are listed in Supplementary Table 1.

### Generation of *V*_*H*_*7-3 Igh* pre-rearranged; *Rag2*^−/−^ mouse model

The heterozygous or homozygous *V*_*H*_*7-3 Igh* pre-rearranged mice (*V*_*H*_*7-3*^*wt/re*^ or *V*_*H*_*7-3*^*re/re*^) were generated through induced pluripotent stem (iPS) cells and maintained in the Alt laboratory. To perform 3C-HTGTS experiments with RAG2-deficient background, *V*_*H*_*7-3*^*wt/re*^ or *V*_*H*_*7-3*^*re/re*^ mice were crossed with *Rag2*^−/−^ mice to obtain *V*_*H*_*7-3*^*wt/re*^*; Rag2*^−/−^ or *V*_*H*_*7-3*^*re/re*^*; Rag2*^−/−^ mice on the 129SV background.

### Purification of bone marrow precursor B cells

For RAG on-target and off-target analysis, single cell suspensions were derived from bone marrows of 4- to 6-week-old male and female wild-type and *Igk* Vκ locus inversion 129SV mice and incubated in Red Blood Cell Lysing Buffer (Sigma-Aldrich, R7757) to deplete the erythrocytes. B220^+^CD43^low^IgM^−^ pre-B cells were isolated by staining with anti-B220–APC (1:1,000 dilution; eBioscience, 17-0452-83), anti-CD43–PE (1:400 dilution; BD Biosciences, 553271) and anti-IgM–FITC (1:500 dilution; eBioscience, 11-5790-81) and purifying via fluorescence-activated cell sorting (FACS), and the purified primary pre-B cells were directly used for HTGTS-V(D)J-seq as described^21,43^.

For 3C-HTGTS experiments, B220-positive primary pre-B cells were purified via anti-B220 MicroBeads (Miltenyi, 130-049-501) from 4- to 6-week-old male and female *V*_*H*_*7-3*^*wt/re*^*; Rag2*^−/−^ or *V*_*H*_*7-3*^*re/re*^*; Rag2*^−/−^ mice. Purified pre-B cells from 3 or 4 mice were pooled together for each 3C-HTGTS experiment. Each mouse was double-checked and confirmed by PCR and Sanger sequencing prior to various assays.

### Generation of single Jκ5 *v-Abl* cell line and its derivatives

The construction of sgRNA–Cas9 plasmids and methods for nucleofection-mediated targeting experiments described for this section and all subsequent paragraphs describing *v-Abl* line modifications were performed as previously described^7^. All *v-Abl* cell lines have not been tested for mycoplasma contamination.

The initial ‘parental’ *Rag2*^−/−^;*Eμ-Bcl2*^+^ *v-Abl* cell line in the 129SV background was generated previously^6^. Random 1–4 bp indels (barcodes) were introduced into a site ~85 bp downstream of the Jκ5-RSS heptamer and ~40 bp upstream of the Jκ5 bait primer on both alleles in this parental line, similarly to the approach previously described to modify J_H_4^6^. The resulting ‘Jκ5-barcoded’ *v-Abl* line was further targeted with sgRNA1 and sgRNA2 to invert the whole Vκ locus on one allele and leaving the other allele intact. Thus, the *Igk* allele-specific barcode permits the separation of sequencing reads derived from the wild-type allele and the Vκ inverted allele assayed with the same bait primer under the same cellular context. This barcoded line was used to generate the data in Fig. 1b,e.

To facilitate further modifications on the *Igk* locus, the Jκ5-barcoded *v-Abl* line was targeted with sgRNA1 and sgRNA3 that deleted the entire *Igk* locus on one allele and left the other allele intact. The barcode was not relevant to further studies based on this single *Igk* allele line or its derivatives. The single *Igk* allele line was further targeted by another two pairs of sgRNAs to separately delete Jκ1 to Jκ4 (sgRNA4 and sgRNA5) and downstream *Igk*-RS (sgRNA6 and sgRNA7) to exclude confounding secondary rearrangements and keep the configuration unchanged between Jκ5 and iEκ. This line is referred to as the ‘single Jκ5 allele line’.

The single Jκ5 allele line was further modified by specifically designed Cas9–sgRNA to generate the single Jκ5-Vκ inv line (sgRNA8 and sgRNA9), single Jκ5-inv line (sgRNA10 and sgRNA11), single Jκ5-single *Igh* line (sgRNA12 and sgRNA13), single Jκ5-PKO line (sgRNA2 and sgRNA14), single Jκ5-*Cer* knockout (KO) line (sgRNA15 and sgRNA16), single Jκ5-*Sis* KO line (sgRNA17 and sgRNA18), and single Jκ5-*CerSis* KO line (sgRNA15 and sgRNA18).

The single Jκ1 allele *v-Abl* line was generated from the single *Igk* allele line by separately deleting Jκ2 to Jκ5 (sgRNA10 and sgRNA19) and deleting downstream *Igk*-RS (sgRNA6 and sgRNA7).

All candidate clones with desired gene modifications were screened by PCR and confirmed by Sanger sequencing.

### Generation and analysis of DJ_H_ pre-rearranged WAPL-degron *v-Abl* cell lines

The DJ_H_ pre-rearranged *v-Abl* lines in C57BL/6 background were derived from the previously described WAPL-degron *v-Abl* line^7^. The open reading frame sequences of *Rag1* and *Rag2* were cloned into pMAX-GFP vector (Addgene, 177825) following the standard protocol to generate pMAX-Rag1 and pMAX-Rag2 plasmids. These two plasmids (each 2.5 μg) were nucleofected into 2.0 × 10^6^ WAPL-degron *v-Abl* cells to allow endogenous D-to-J_H_ rearrangements mediated by transient RAG expression. Cells harbouring the desired DQ52J_H_4 rearrangement (DJ_H_-WT line) were subsequently identified by PCR screening and verified by Sanger sequencing. The DJ_H_-inv *v-Abl* line was generated from the DJ_H_-WT line by using Cas9–sgRNA to target sequences downstream of J_H_4 and upstream of DQ52 (sgRNA20 and sgRNA21). The DJ_H_-WT and DJ_H_-inv lines were treated with IAA and Dox to deplete WAPL as described^7^.

### Generation of *Igh–Igk* hybrid *v-Abl* cell line and its derivatives

The *Igh–Igk* hybrid *v-Abl* cell line was derived from the single Jκ5 allele *v-Abl* line. In brief, the single Jκ5 allele line was targeted by sgRNA12 and sgRNA13 to generate the single Jκ5-single *Igh* line where the entire *Igh* locus was deleted from one allele. The single Jκ5-single *Igh* line was then targeted by sgRNA22 (cut 1, upstream of IGCR1 in *Igh*) and sgRNA8 (cut 2, upstream of Vκ2-137 in *Igk*) to generate a balanced chromosomal translocation between chromosomes 12 and 6. In the resulting *Igh–Igk* hybrid *v-Abl* line, the entire *Igk* locus along with the rest of chromosome 6 was appended onto chromosome 12 at a point upstream of IGCR1 in *Igh*, and the *Igh* V_H_ locus along with the small telomeric portion of chromosome 12 was reciprocally appended onto chromosome 6. To generate the *Igh–Igk* hybrid-Vκ line, the *Igh–Igk* hybrid line was sequentially modified to invert the entire Vκ locus (sgRNA15 and sgRNA23), mutate DQ52 RSSs (sgRNA24 and ssODN1) and delete all upstream D segments (sgRNA25 and sgRNA26). To generate the *Igh–Igk* hybrid-Vκ-JκRSS-PKO line from the *Igh–Igk* hybrid-Vκ-JκRSS line, sgRNA2 and sgRNA14 were used to delete the proximal Vκ domain.

To generate the *Igh–Igk* hybrid-D-J_H_ line, the *Igh–Igk* hybrid line was targeted by sgRNA27 and sgRNA28 to delete IGCR1 and the entire Vκ locus. The *Igh–Igk* hybrid-D-J_H_ line was further modified to generate the *Igh–Igk* hybrid-D line where J_H_1-4 has been deleted (sgRNA29 and sgRNA30).

All candidate clones with desired gene modifications were screened by PCR and confirmed by Sanger sequencing. See Fig. 4a and Extended Data Figs. 5a and 8a for detailed strategy and procedure.

### Whole-chromosome painting

Whole-chromosome painting was performed on single Jκ5-single *Igh v-Abl* line and *Igh–Igk* hybrid *v-Abl* line using fluorescent probes tiling the entire chromosome 6 (Chr6-FITC, Applied Spectral Imaging) and chromosome 12 (Chr12-TxRed, Applied Spectral Imaging) according to standard protocol. In brief, cells were treated with colcemid at 0.05 μg ml^−1^ final concentration for 3 h before being processed for metaphase drop. The slides were dehydrated in ethanol series, denatured at 70 °C for 1.5 min, and hybridized to denatured probe mixture at 37 °C for 12–16 h. The slides were then washed, stained with DAPI, and imaged with Olympus BX61 microscope. ImageJ (1.53q) was used for image processing.

### RSS replacement experiments

All RSS replacement modifications were generated via Cas9–sgRNA using short single-stranded DNA oligonucleotide (ssODN) as donor template. In brief, 2.5 μg Cas9–sgRNA plasmid and 5 μl 10 μM ssODN were co-transfected into 2.0 × 10^6^ *v-Abl* cells. PCR screening was performed sequentially on pooled clones and then single clones, and subsequently verified by Sanger sequencing. Specifically, sgRNA31 and ssODN2 were used to replace J_H_1-RSS with Jκ5-RSS in *Igh–Igk* hybrid-Vκ *v-Abl* line to generate the *Igh–Igk* hybrid-Vκ-JκRSS line; sgRNA32 and ssODN3 were used to replace Jκ5-RSS with J_H_1-RSS in single Jκ5-single *Igh* line to generate the single Jκ5-single *Igh*-J_H_RSS line; sgRNA33 and ssODN4 were used to replace DQ52 upstream RSS with Vκ12-44-RSS in *Igh–Igk* hybrid-D line to generate the *Igh–Igk* hybrid-D-VκRSS line; sgRNA34 and ssODN5 were used to replace DQ52 upstream RSS with Vκ11-125-RSS in DJ_H_-inv line to generate the DJ_H_-inv-VκRSS line; sgRNA35 and ssODN6 were used to replace V_H_5-2-RSS with Jκ1-RSS in DJ_H_-inv-VκRSS line to generate the DJ_H_-inv-VκRSS-JκRSS line.

### RAG complementation

RAG was reconstituted in RAG1-deficient *v-Abl* cells via retroviral infection with the pMSCV-RAG1-IRES-Bsr and pMSCV-Flag-RAG2-GFP vectors followed by 3–4 days of blasticidin (Sigma-Aldrich, 15205) selection to enrich for cells with virus integration^7^. RAG2 was reconstituted in RAG2-deficient *v-Abl* cells via retroviral infection with the pMSCV-Flag-RAG2-GFP vector followed by two days of puromycin (ThermoFisher, J67236) selection to enrich for cells with virus integration^5^.

### HTGTS-V(D)J-seq and data analyses

HTGTS-V(D)J-seq libraries were prepared as previously described^6,7,21,43^ with 0.5–2 μg of genomic DNA (gDNA) from sorted primary pre-B cells or 10 μg of gDNA from G1-arrested RAG-complemented RAG-deficient *v-Abl* cells. The final libraries were sequenced on Illumina NextSeq550 with control software (2.2.0) or NextSeq2000 with control software (1.5.0.42699) using paired-end 150-bp sequencing kit. HTGTS-V(D)J-seq libraries were processed via the pipeline described previously^43^. For *Igh* rearrangement analysis in DJ_H_-WT and DJ_H_-inv WAPL-degron *v-Abl* lines, the data were aligned to the mm9_DQ52J_H_4 genome and analysed with all duplicate junctions included in the analyses as previously described^43^. For analysis in DJ_H_-inv-VκRSS and DJ_H_-inv-VκRSS-JκRSS *v-Abl* lines, the data were aligned to the mm9_DQ52J_H_4_VκRSS genome. For all other rearrangement analysis, primary pre-B cells and *v-Abl* cells used are from 129SV background. Since there is almost no difference in the *Igk* locus between C57BL/6 and 129SV genomic backgrounds^44^, the data were aligned to the AJ851868/mm9 hybrid (mm9AJ) genome^6^ except: data from *Igh–Igk* hybrid-Vκ-JκRSS and *Igh–Igk* hybrid-Vκ-JκRSS-PKO *v-Abl* lines were aligned to the mm9AJ_J_H_1toJκ5RSS genome, data from single Jκ5-single *Igh*-J_H_RSS *v-Abl* line were aligned to the mm9AJ_Jκ5toJ_H_1RSS genome, and data from *Igh–Igk* hybrid-D-VκRSS *v-Abl* line were aligned to the mm9AJ_DQ52uptoVκRSS genome. To show the absolute level of V(D)J recombination, each HTGTS-V(D)J-seq library was down-sampled to 500,000 total reads (junctions + germline reads); to show the relative Vκ usage pattern across the Vκ locus, individual Vκ usage levels were divided by the total Vκ usage level in each HTGTS-V(D)J-seq library to obtain the relative percentage. Such analyses are useful for examining effects of potential regulatory element mutations. For example, differences in absolute rearrangement levels between two samples with the same relative rearrangement patterns would reflect differences in RAG or RC activity without changes in long-range regulatory mechanisms^7,26^.

RAG off-targets were extracted from corresponding normalized HTGTS-V(D)J-seq libraries by removing on-target junctions on bona fide RSSs. We noticed the remaining junctions in the *Igk* locus were skewed to a few very strong RSS sites, which represent unannotated bona fide RSSs not associated with functional Vκ segments. We eliminated these strong RSSs from our cryptic RSS analyses by filtering out RSS sites with a CAC and additional at least 9 bp matches to the remaining ideal heptamer AGTG and ideal nonamer ACAAAAACC in the context of a 12-or-23-bp spacer—that is, at most 4-bp mismatches to the ideal RSS site. In addition, because coding end junctions are processed and can spread across several bps beyond the CAC cleavage site^4^, the new code has the advantage of collapsing these coding end junctional signals within 15 bp into one peak mapped to the CAC cleavage site for better visualization of off-target coding junction peaks. For visualization of the actual distribution of coding end junctions, one can reveal them through analysis with our prior pipeline. Details of both pipelines used are provided in Code availability. Junctions are denoted as deletional if the prey cryptic RSS is in convergent orientation with the bait RSS and as inversional if the prey cryptic RSS is in the same orientation with the bait RSS.

### 3C-HTGTS and data analyses

3C-HTGTS was performed as previously described^3^ on G1-arrested RAG2-deficient *v-Abl* cells^3,5–7,26^. Reference genomes were the same as used in HTGTS-V(D)J-seq data analyses described above. To better normalize 3C-HTGTS libraries and reduce the impact of the level of self-ligation (circularization), the high peaks upstream of the bait site were filtered out, following the same rationale as described for 4C-seq^45^. For iEκ-baited 3C-HTGTS libraries, we removed bait site peaks in the chr. 6:70,675,300–70,675,450 region; For *Cer* CBE1-baited 3C-HTGTS libraries, we removed bait site peaks in chr. 6:70,659,550–70,659,700 region; For *Sis* CBE2-baited 3C-HTGTS libraries, we removed bait site peaks in chr. 6:70,664,600–70,664,800 region; For IGCR1 CBE1-baited 3C-HTGTS libraries, we removed bait site peaks in the chr12:114,740,239–114,740,353 region. Then, only the junctions inside of a genomic region (chr. 6:64,515,000–73,877,000 for the entire *Igk* locus; chr. 12:111,453,935–120,640,000 for the entire *Igh* locus; chr. 6:64,515,000–70,658,827 and chr. 12:111,453,935-114,824,843 for the *Igh–Igk* hybrid-Vκ locus) encompassing the entire Ig locus were retained (see details in Code availability). After processing as described above, the retained junctions of the 3C-HTGTS libraries were further normalized to 50,827 total number of junctions, which is the junction number recovered from the smallest library in the set of libraries being compared. The sequences of primers used for generating 3C-HTGTS libraries are listed in Supplementary Table 1.

Unlike ChIP-seq, the junctions of 3C-HTGTS data are discontinuously distributed on the genome, but mainly on the enzyme cutting sites (CATG by NlaIII). To call peaks for 3C-HTGTS data, we first collapsed the junction signals to nearby enzyme cutting sites, and discarded signals far away (>10 bp) from enzyme cutting sites. Then, we only focused on the cutting sites with signals, calculated the median with a moving window of 101 cutting sites (one centre, 50 left, and 50 right sites). We did a Poisson test for each site, with the median as a conservative over-estimation of the lambda parameter of Poisson distribution. Based on the raw *P* values from the Poisson test, we calculated Bonferroni-adjusted *P* values, called peak summits at the sites with adjusted *P* value < 0.05, and determined the range of peak region by progressively extending the two sides to the sites that have local maximum raw *P* value and also the raw *P* values ≥ 0.05. Nearby overlapping peak regions were merged as one peak region, and only the ‘best’ (defined by lowest *P* value) summit was kept after merging. Finally, for each group of multiple repeats, we merged the overlapping peak regions from all repeats, and counted the number of supporting repeats for each merged peak region. We defined and only kept the ‘robust’ peak regions that were supported by >50% of the repeats (that is, ≥ 2 supporting repeats among 2 or 3 repeats, or ≥ 3 supporting repeats among 4 or 5 repeats), and the ‘best’ (defined by lowest *P* value) summit information was reported.

We further annotated and quantified the features underlying each of the robust 3C-HTGTS peak region ±1 kb. We focused on CBEs, E2A-binding sites, and transcription. For CBEs, we first scanned the possible CBEs by MEME-FIMO using the CTCF motif record (MA0139.1) in JASPAR 2018 core vertebrate database. We applied MACS2 to call peaks in the three repeats of published CTCF ChIP-seq data in parental *v-Abl* line^6^, and only kept ‘reliable’ CBEs with motif score > 13 and overlapping with peaks called in ≥2 repeats. We counted the number of reliable CBEs within each of the robust 3C-HTGTS peak region ±1 kb, and defined them as having an underlying CBE if the number ≥ 1. For E2A-binding sites, we applied MACS2 to get the signal bigwig file from the published E2A ChIP-seq data^46^, and then annotated the maximum E2A ChIP-seq signal value within each of the robust 3C-HTGTS peak region ±1 kb. We defined peaks having underlying E2A site if the maximum signal ≥ 0.5. For transcription, we annotated the maximum and the average signal of the three repeats of published GRO-seq data in parental *v-Abl* line^6^, and defined a peak as having transcription if the maximum signal ≥40 or the average signal ≥10 in ≥2 repeats. See details in Code availability.

### Quantification and statistical analysis

Graphs were generated using GraphPad Prism 10, Origin 2023b and R version 3.6.3. After normalization in each sample, 3C-HTGTS, ChIP-seq and GRO-seq signals of multiple repeats were merged as mean ± s.e.m. of the maximum value in each repeat in each bin, after dividing the plotting region into 1,000 bins (Fig. 2m and Extended Data Fig. 2) or 200 bins (Supplementary Data 1). Unpaired, two-sided Welch’s *t*-test was used to compare total rearrangement levels between indicated samples, with *P* values presented in relevant figure legends. Pearson correlation coefficient (*r*) and the corresponding *P* value were calculated to determine the similarity in Vκ usage pattern between indicated samples after calculating the average usage among repeats, and are presented in relevant figure legends.

### Availability of materials

All plasmids, cell lines and mouse lines generated in this study are available from the authors upon request.

### Reporting summary

Further information on research design is available in the Nature Portfolio Reporting Summary linked to this article.

## Online content

Any methods, additional references, Nature Portfolio reporting summaries, source data, extended data, supplementary information, acknowledgements, peer review information; details of author contributions and competing interests; and statements of data and code availability are available at 10.1038/s41586-024-07477-y.

### Supplementary information

Supplementary Data 1 *Cer*-bait 3C-HTGTS interaction peaks and underlying features in the Vκ locus from RAG-deficient primary pre-B cells and *v-Abl* cells. Related to Fig. 2m and Extended Data Fig. 2b. Zoom-in profiles of *Cer*-bait 3C-HTGTS in RAG2-deficient primary pre-B cells (first lane, pink) and RAG2-deficient *v-Abl* cells (second lane, blue) were shown along with annotated CBE motifs (third lane, red for rightward CBE, blue for leftward CBE), CTCF ChIP-seq (fourth lane, red), Rad21 ChIP-seq (fifth lane, blue), E2A ChIP-seq (sixth lane, purple) and GRO-seq (bottom lane, red for rightward transcription, blue for leftward transcription) within ±10kb region of all peaks called in either primary pre-B cells or *v-Abl* cells. For each peak, underlying features within ±1kb are indicated, including rightward CBE (“C” in red), leftward CBE (“C” in blue), E2A binding sequence (“E”) and transcription (“T”). Peaks without any obvious underlying features are labeled as unknown (“U”). Vκ domains are indicated by blue (distal), gray (middle) and orange (proximal) shadows. CBE annotation, CTCF ChIP-seq, Rad21 ChIP-seq and GRO-seq were replotted from published data in RAG-deficient *v-Abl* cells^6^. E2A ChIP-seq was replotted from published data in RAG-deficient primary pro-B cells^46^. See Methods for more details on 3C-HTGTS peak calling and underlying feature analysis. Data are presented as mean value from 2 biological repeats (3C-HTGTS) or as mean ± s.e.m. from 3 biological repeats (CTCF ChIP-seq, Rad21 ChIP-seq and GRO-seq).

Reporting Summary

Supplementary Table 1 sgRNAs, oligonucleotides and primers used in this study.

### Source data

Source Data Fig. 1

Source Data Fig. 2

Source Data Fig. 3

Source Data Fig. 4

Source Data Fig. 5

Source Data Extended Data Fig. 1

Source Data Extended Data Fig. 3

Source Data Extended Data Fig. 4

Source Data Extended Data Fig. 5

Source Data Extended Data Fig. 6

Source Data Extended Data Fig. 7

Source Data Extended Data Fig. 8

## Data Availability

High-throughput sequencing data reported in this study have been deposited in the Gene Expression Omnibus (GEO) database under the accession number GSE263124, with subseries GSE254039 for HTGTS-V(D)J-seq data and GSE263123 for 3C-HTGTS data. The consensus CTCF-binding motif was extracted from JASPAR 2018 core vertebrate database (http://jaspar2018.genereg.net/matrix/MA0139.1). Source data are provided with this paper.

## Code availability

HTGTS-V(D)J-seq and 3C-HTGTS data were processed through published pipelines as previously described^43^. Specifically, the pipelines analysing HTGTS data are available at http://robinmeyers.github.io/transloc_pipeline/. Newly developed pipelines for off-targets filtering on cryptic RSS and 3C-HTGTS normalization and peak calling are available at https://github.com/Yyx2626/HTGTS_related.
