# Supplementary material for: Molecular basis for differential Igk versus Igh V(D)J joining mechanisms
Source: Nature. 2024 May 29;630(8015):189–97. doi: 10.1038/s41586-024-07477-y (PMC11153149; doi:10.1038/s41586-024-07477-y)
Supplement: Supplementary file 1 — Cer-bait 3C-HTGTS interaction peaks and underlying features in the Vκ locus from RAG-deficient primary pre-B cells and v-Abl cells. Related to Fig. 2m and Extended Data Fig. 2b. Zoom-in profiles of Cer-bait 3C-HTGTS in RAG2-deficient primary pre-B cells (first lane, pink) and RAG2-deficient v-Abl cells (second lane, blue) were shown along with annotated CBE motifs (third lane, red for rightward CBE, blue for leftward CBE), CTCF ChIP-seq (fourth lane, red), Rad21 ChIP-seq (fifth lane, blue), E2A ChIP-seq (sixth lane, purple) and GRO-seq (bottom lane, red for rightward transcription, blue for leftward transcription) within ±10kb region of all peaks called in either primary pre-B cells or v-Abl cells. For each peak, underlying features within ±1kb are indicated, including rightward CBE (“C” in red), leftward CBE (“C” in blue), E2A binding sequence (“E”) and transcription (“T”). Peaks without any obvious underlying features are labeled as unknown (“U”). Vκ domains are indicated by blue (distal), gray (middle) and orange (proximal) shadows. CBE annotation, CTCF ChIP-seq, Rad21 ChIP-seq and GRO-seq were replotted from published data in RAG-deficient v-Abl cells6. E2A ChIP-seq was replotted from published data in RAG-deficient primary pro-B cells46. See Methods for more details on 3C-HTGTS peak calling and underlying feature analysis. Data are presented as mean value from 2 biological repeats (3C-HTGTS) or as mean ± s.e.m. from 3 biological repeats (CTCF ChIP-seq, Rad21 ChIP-seq and GRO-seq). [file 41586_2024_7477_MOESM1_ESM.pdf]

nature portfolio

https://doi.org/10.1038/s41586-024-07477-y

Supplementary information

Molecular basis for differential *Igk* versus *Igh* V(D)J joining mechanisms

In the format provided by the
authors and unedited

Supplementary Data 1 | ***Cer*-bait 3C-HTGTS interaction peaks and underlying features in the Vκ locus from RAG-deficient primary pre-B cells and *v-Abl* cells.**

Related to Fig.2m and Extended Data Fig.2b. Zoom-in profiles of *Cer*-bait 3C-HTGTS in RAG2-deficient primary pre-B cells (first lane, pink) and RAG2-deficient *v-Abl* cells (second lane, blue) were shown along with annotated CBE motifs (third lane, red for rightward CBE, blue for leftward CBE), CTCF ChIP-seq (fourth lane, red), Rad21 ChIP-seq (fifth lane, blue), E2A ChIP-seq (sixth lane, purple) and GRO-seq (bottom lane, red for rightward transcription, blue for leftward transcription) within ± 10kb region of all peaks called in either primary pre-B cells or *v-Abl* cells. For each peak, underlying features within ± 1kb are indicated, including rightward CBE (“C” in red), leftward CBE (“C” in blue), E2A binding sequence (“E”) and transcription (“T”). Peaks without any obvious underlying features are labeled as unknown (“U”). Vκ domains are indicated by blue (distal), gray (middle) and orange (proximal) shadows. CBE annotation, CTCF ChIP-seq, Rad21 ChIP-seq and GRO-seq were replotted from published data in RAG-deficient *v-Abl* cells (Ba et al., Nature 2020). E2A ChIP-seq was replotted from published data in RAG-deficient primary pro-B cells (Lin et al., Nat. Immunol. 2010). See Methods for more details on 3C-HTGTS peak calling and underlying feature analysis. Data are presented as mean value from 2 biological repeats (3C-HTGTS) or as mean ± s.e.m. from 3 biological repeats (CTCF ChIP-seq, Rad21 ChIP-seq and GRO-seq).

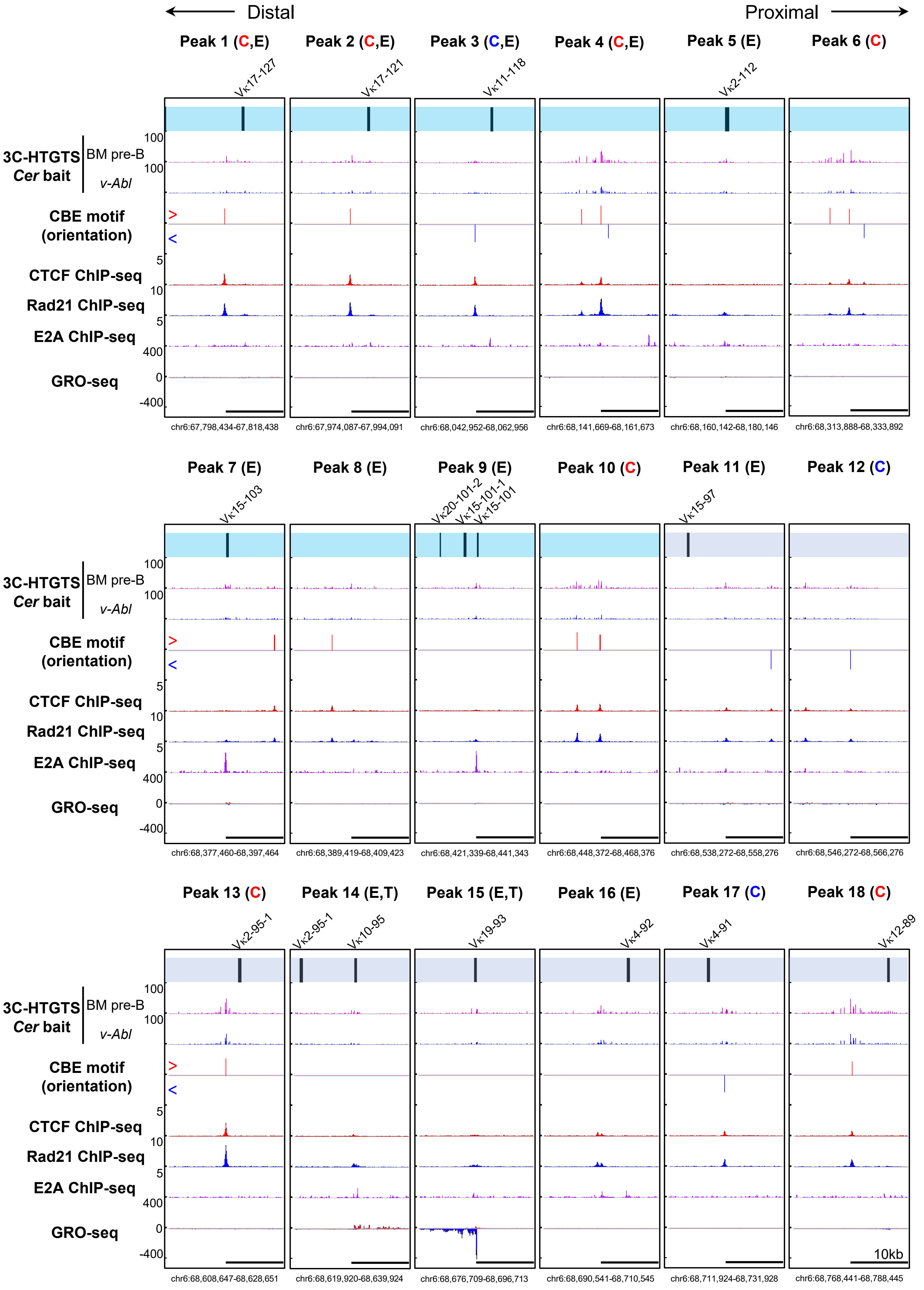

Distal
Proximal
Peak 1 (C,E)
Peak 2 (C,E)
Peak 3 (C,E)
Peak 4 (C,E)
Peak 5 (E)
Peak 6 (C)
Vκ17-127
Vκ17-121
Vκ11-118
Vκ2-112
3C-HTGTS Cer bait
BM pre-B
v-Abl
CBE motif (orientation)
CTCF ChIP-seq
Rad21 ChIP-seq
E2A ChIP-seq
GRO-seq
chr6:67,798,434-67,818,438
chr6:67,974,087-67,994,091
chr6:68,042,952-68,062,956
chr6:68,141,669-68,161,673
chr6:68,160,142-68,180,146
chr6:68,313,888-68,333,892
Peak 7 (E)
Peak 8 (E)
Peak 9 (E)
Peak 10 (C)
Peak 11 (E)
Peak 12 (C)
Vκ15-103
Vκ20-101-2
Vκ15-101-1
Vκ15-101
Vκ15-97
chr6:68,377,460-68,397,464
chr6:68,389,419-68,409,423
chr6:68,421,339-68,441,343
chr6:68,448,372-68,468,376
chr6:68,538,272-68,558,276
chr6:68,546,272-68,566,276
Peak 13 (C)
Peak 14 (E,T)
Peak 15 (E,T)
Peak 16 (E)
Peak 17 (C)
Peak 18 (C)
Vκ2-95-1
Vκ10-95
Vκ19-93
Vκ4-92
Vκ4-91
Vκ12-89
10kb
chr6:68,608,647-68,628,651
chr6:68,619,920-68,639,924
chr6:68,676,709-68,696,713
chr6:68,690,541-68,710,545
chr6:68,711,924-68,731,928
chr6:68,768,441-68,788,445

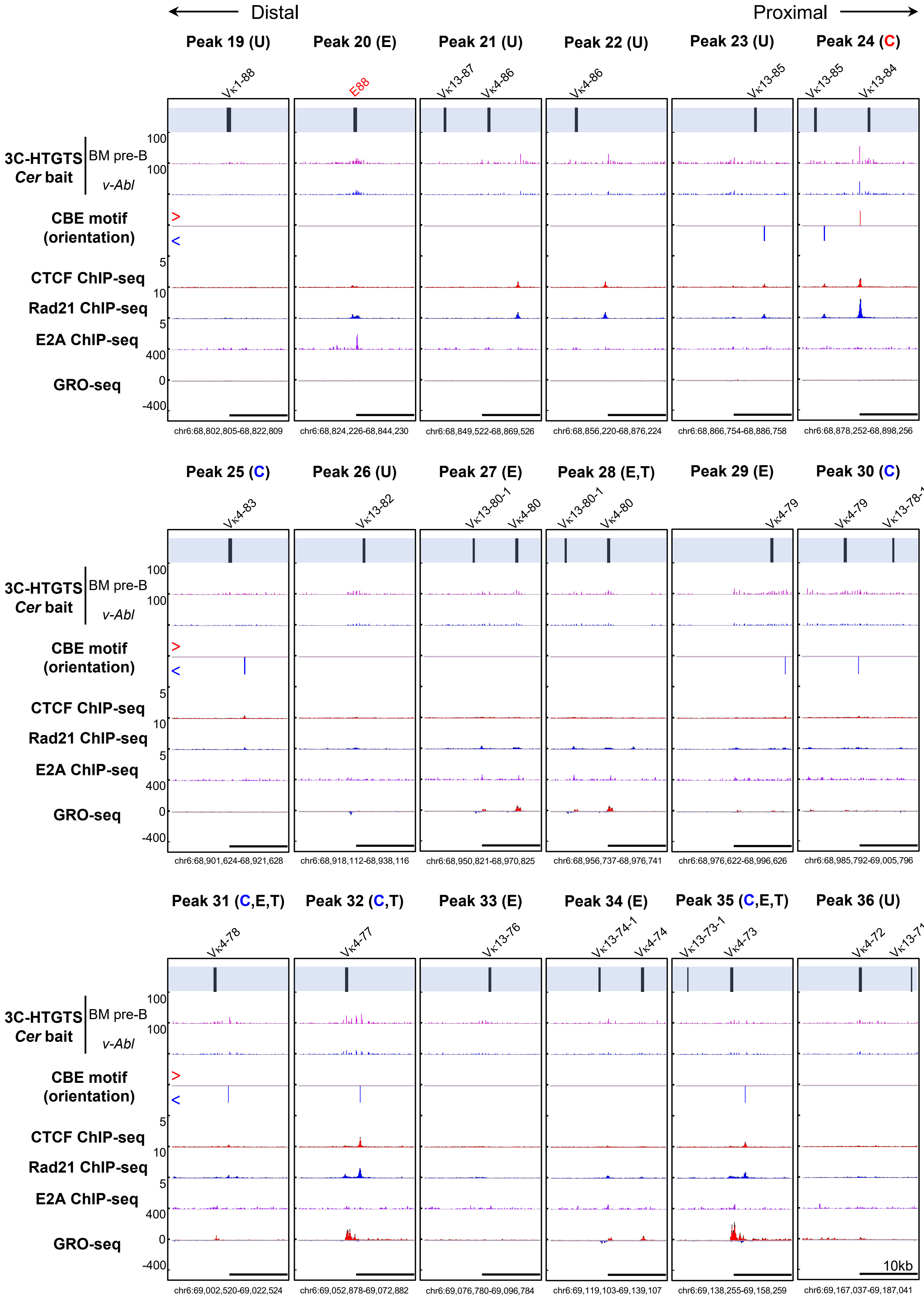

Distal
Proximal
Peak 19 (U)
Peak 20 (E)
Peak 21 (U)
Peak 22 (U)
Peak 23 (U)
Peak 24 (C)
Vκ1-88
E88
Vκ13-87
Vκ4-86
Vκ4-86
Vκ13-85
Vκ13-85
Vκ13-84
3C-HTGTS
Cer bait
BM pre-B
v-Abl
CBE motif
(orientation)
CTCF ChIP-seq
Rad21 ChIP-seq
E2A ChIP-seq
GRO-seq
chr6:68,802,805-68,822,809
chr6:68,824,226-68,844,230
chr6:68,849,522-68,869,526
chr6:68,856,220-68,876,224
chr6:68,866,754-68,886,758
chr6:68,878,252-68,898,256
Peak 25 (C)
Peak 26 (U)
Peak 27 (E)
Peak 28 (E,T)
Peak 29 (E)
Peak 30 (C)
Vκ4-83
Vκ13-82
Vκ13-80-1
Vκ4-80
Vκ13-80-1
Vκ4-80
Vκ4-79
Vκ4-79
Vκ13-78-1
chr6:68,901,624-68,921,628
chr6:68,918,112-68,938,116
chr6:68,950,821-68,970,825
chr6:68,956,737-68,976,741
chr6:68,976,622-68,996,626
chr6:68,985,792-69,005,796
Peak 31 (C,E,T)
Peak 32 (C,T)
Peak 33 (E)
Peak 34 (E)
Peak 35 (C,E,T)
Peak 36 (U)
Vκ4-78
Vκ4-77
Vκ13-76
Vκ13-74-1
Vκ4-74
Vκ13-73-1
Vκ4-73
Vκ4-72
Vκ13-71-1
10kb
chr6:69,002,520-69,022,524
chr6:69,052,878-69,072,882
chr6:69,076,780-69,096,784
chr6:69,119,103-69,139,107
chr6:69,138,255-69,158,259
chr6:69,167,037-69,187,041

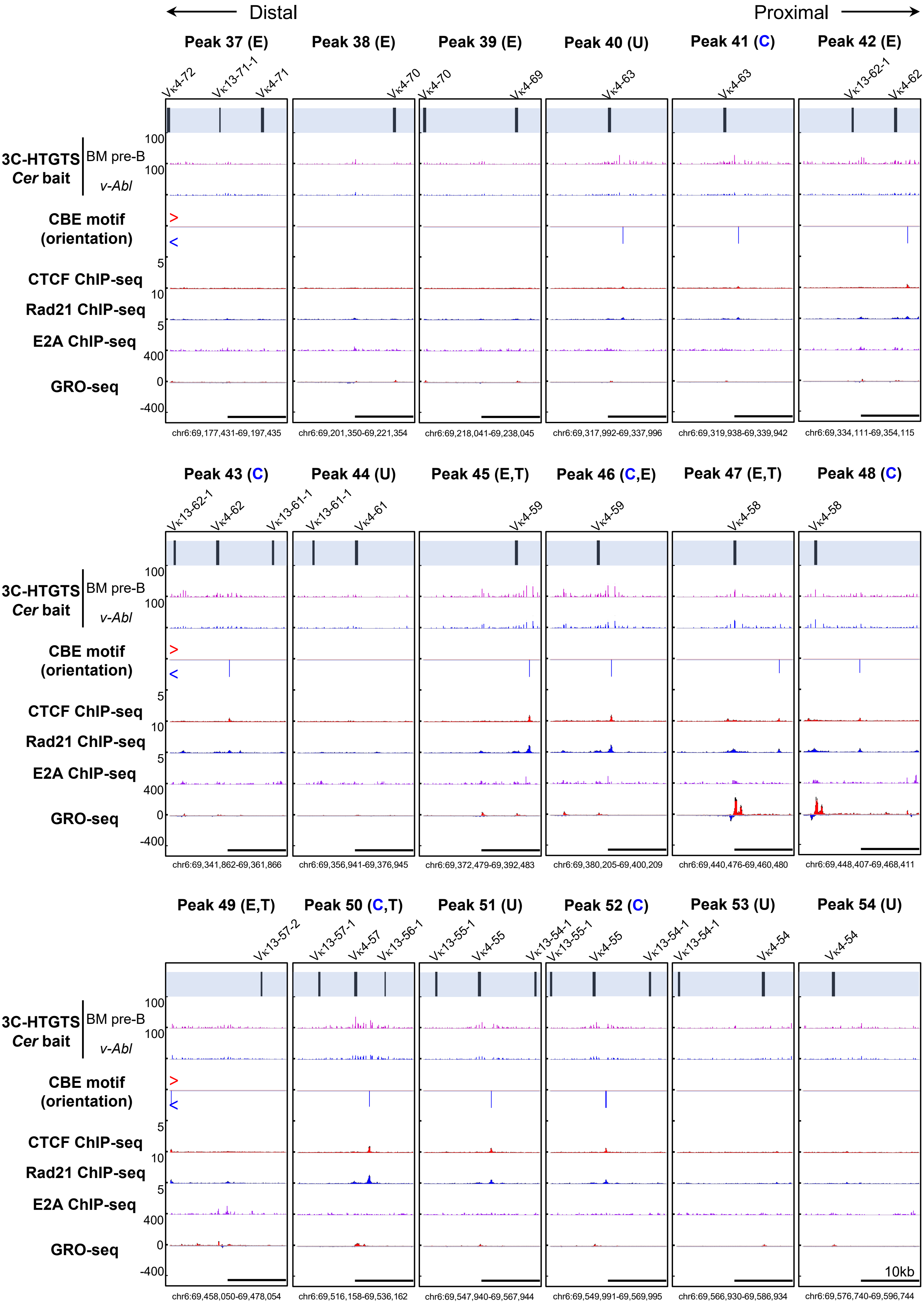

Distal
Proximal
Peak 37 (E)
Peak 38 (E)
Peak 39 (E)
Peak 40 (U)
Peak 41 (C)
Peak 42 (E)
3C-HTGTS Cer bait
BM pre-B
v-Abl
CBE motif (orientation)
CTCF ChIP-seq
Rad21 ChIP-seq
E2A ChIP-seq
GRO-seq
chr6:69,177,431-69,197,435
chr6:69,201,350-69,221,354
chr6:69,218,041-69,238,045
chr6:69,317,992-69,337,996
chr6:69,319,938-69,339,942
chr6:69,334,111-69,354,115
Peak 43 (C)
Peak 44 (U)
Peak 45 (E,T)
Peak 46 (C,E)
Peak 47 (E,T)
Peak 48 (C)
chr6:69,341,862-69,361,866
chr6:69,356,941-69,376,945
chr6:69,372,479-69,392,483
chr6:69,380,205-69,400,209
chr6:69,440,476-69,460,480
chr6:69,448,407-69,468,411
Peak 49 (E,T)
Peak 50 (C,T)
Peak 51 (U)
Peak 52 (C)
Peak 53 (U)
Peak 54 (U)
10kb
chr6:69,458,050-69,478,054
chr6:69,516,158-69,536,162
chr6:69,547,940-69,567,944
chr6:69,549,991-69,569,995
chr6:69,566,930-69,586,934
chr6:69,576,740-69,596,744

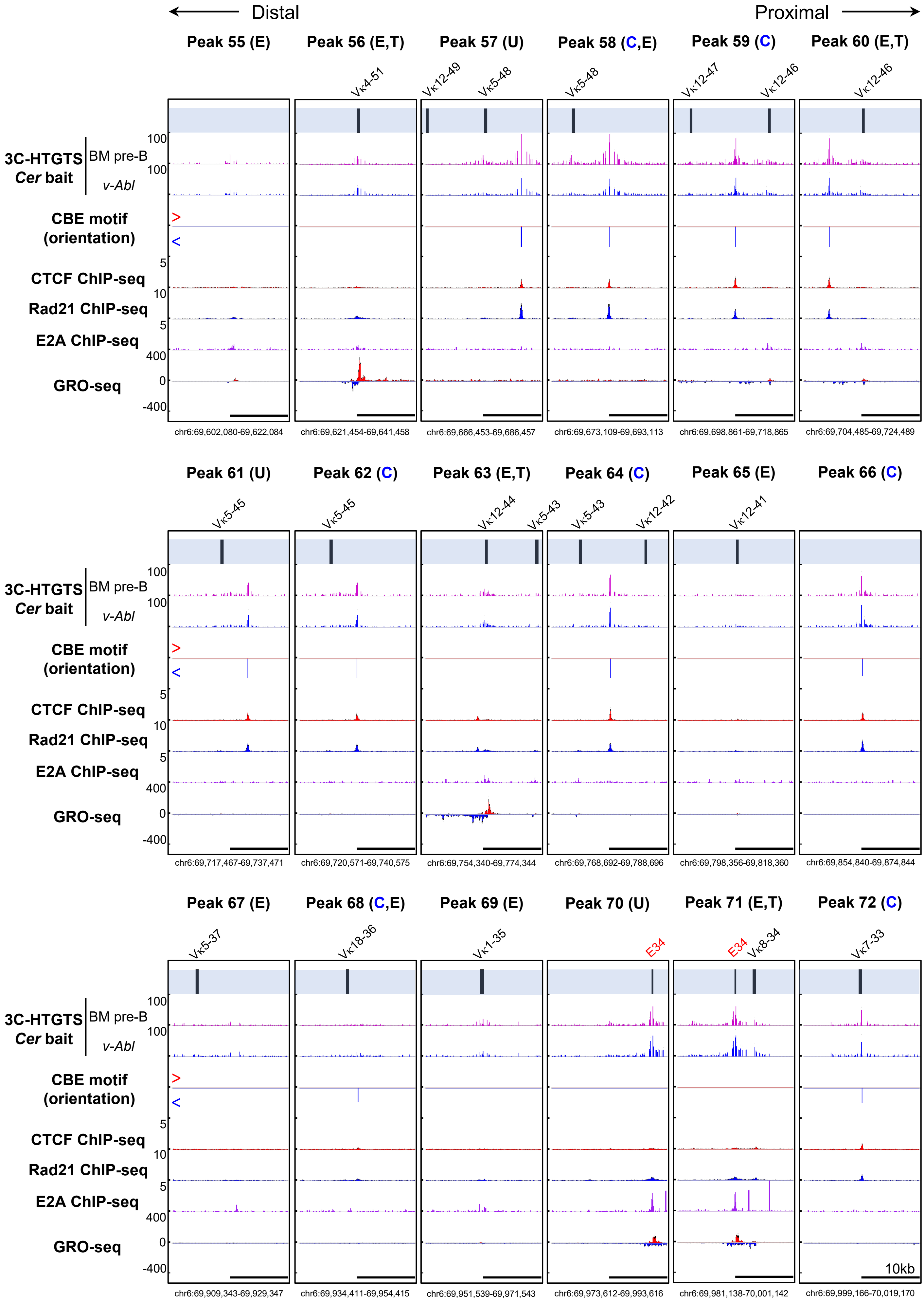
Distal
Proximal
Peak 55 (E)
Peak 56 (E,T)
Peak 57 (U)
Peak 58 (C,E)
Peak 59 (C)
Peak 60 (E,T)
Vκ4-51
Vκ12-49
Vκ5-48
Vκ5-48
Vκ12-47
Vκ12-46
Vκ12-46
3C-HTGTS
Cer bait
BM pre-B
v-Abl
CBE motif
(orientation)
CTCF ChIP-seq
Rad21 ChIP-seq
E2A ChIP-seq
GRO-seq
100
100
5
10
5
400
0
-400
chr6:69,602,080-69,622,084
chr6:69,621,454-69,641,458
chr6:69,666,453-69,686,457
chr6:69,673,109-69,693,113
chr6:69,698,861-69,718,865
chr6:69,704,485-69,724,489
Peak 61 (U)
Peak 62 (C)
Peak 63 (E,T)
Peak 64 (C)
Peak 65 (E)
Peak 66 (C)
Vκ5-45
Vκ5-45
Vκ12-44
Vκ5-43
Vκ5-43
Vκ12-42
Vκ12-41
chr6:69,717,467-69,737,471
chr6:69,720,571-69,740,575
chr6:69,754,340-69,774,344
chr6:69,768,692-69,788,696
chr6:69,798,356-69,818,360
chr6:69,854,840-69,874,844
Peak 67 (E)
Peak 68 (C,E)
Peak 69 (E)
Peak 70 (U)
Peak 71 (E,T)
Peak 72 (C)
Vκ5-37
Vκ18-36
Vκ1-35
E34
E34
Vκ8-34
Vκ7-33
10kb
chr6:69,909,343-69,929,347
chr6:69,934,411-69,954,415
chr6:69,951,539-69,971,543
chr6:69,973,612-69,993,616
chr6:69,981,138-70,001,142
chr6:69,999,166-70,019,170

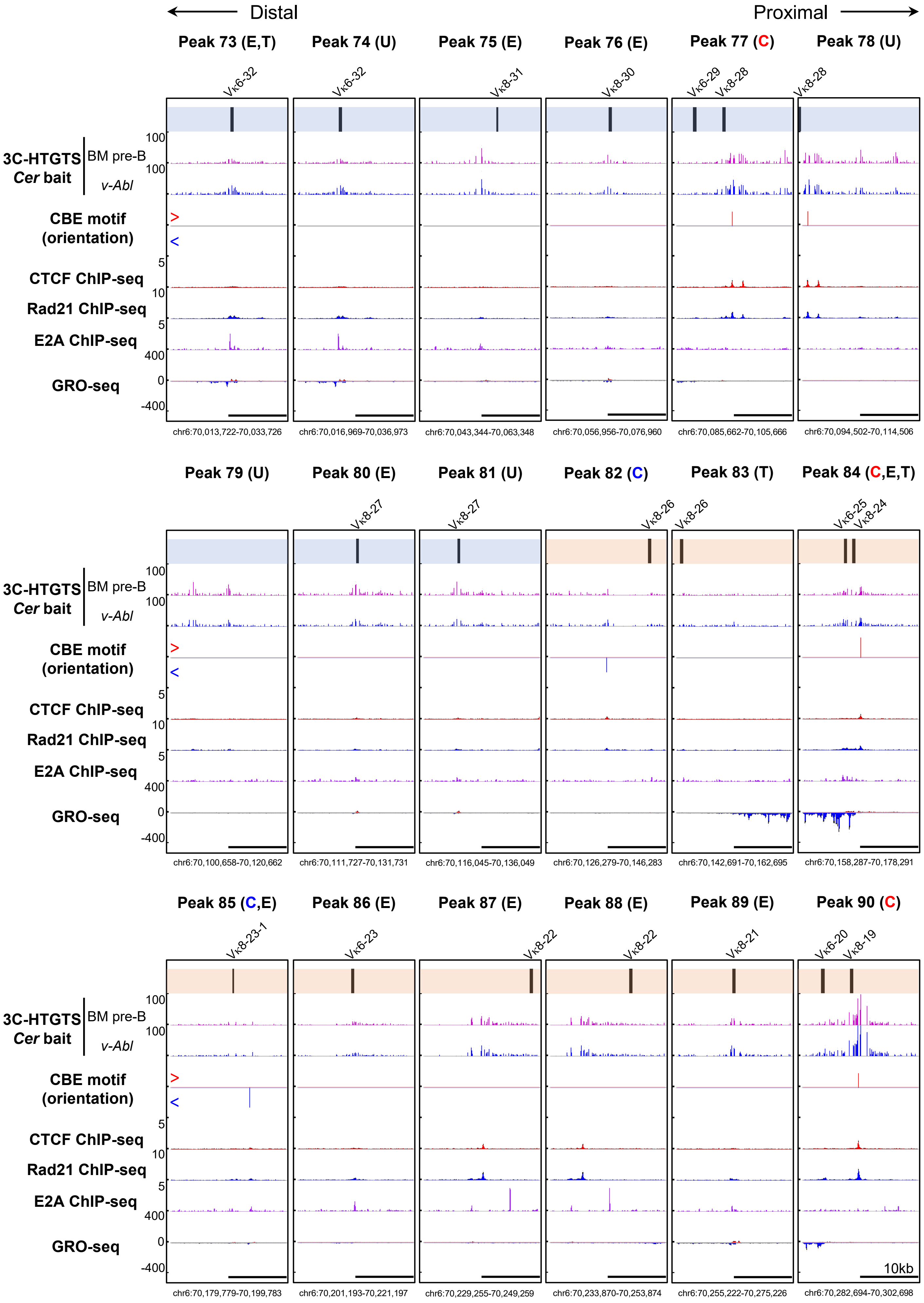

Distal
Proximal
Peak 73 (E,T)
Peak 74 (U)
Peak 75 (E)
Peak 76 (E)
Peak 77 (C)
Peak 78 (U)
Vκ6-32
Vκ6-32
Vκ8-31
Vκ8-30
Vκ6-29
Vκ8-28
Vκ8-28
3C-HTGTS Cer bait
BM pre-B
v-Abl
CBE motif (orientation)
>
<
CTCF ChIP-seq
Rad21 ChIP-seq
E2A ChIP-seq
GRO-seq
100
100
5
10
5
400
0
-400
chr6:70,013,722-70,033,726
chr6:70,016,969-70,036,973
chr6:70,043,344-70,063,348
chr6:70,056,956-70,076,960
chr6:70,085,662-70,105,666
chr6:70,094,502-70,114,506
Peak 79 (U)
Peak 80 (E)
Peak 81 (U)
Peak 82 (C)
Peak 83 (T)
Peak 84 (C,E,T)
Vκ8-27
Vκ8-27
Vκ8-26
Vκ8-26
Vκ6-25
Vκ8-24
chr6:70,100,658-70,120,662
chr6:70,111,727-70,131,731
chr6:70,116,045-70,136,049
chr6:70,126,279-70,146,283
chr6:70,142,691-70,162,695
chr6:70,158,287-70,178,291
Peak 85 (C,E)
Peak 86 (E)
Peak 87 (E)
Peak 88 (E)
Peak 89 (E)
Peak 90 (C)
Vκ8-23-1
Vκ6-23
Vκ8-22
Vκ8-22
Vκ8-21
Vκ6-20
Vκ8-19
10kb
chr6:70,179,779-70,199,783
chr6:70,201,193-70,221,197
chr6:70,229,255-70,249,259
chr6:70,233,870-70,253,874
chr6:70,255,222-70,275,226
chr6:70,282,694-70,302,698

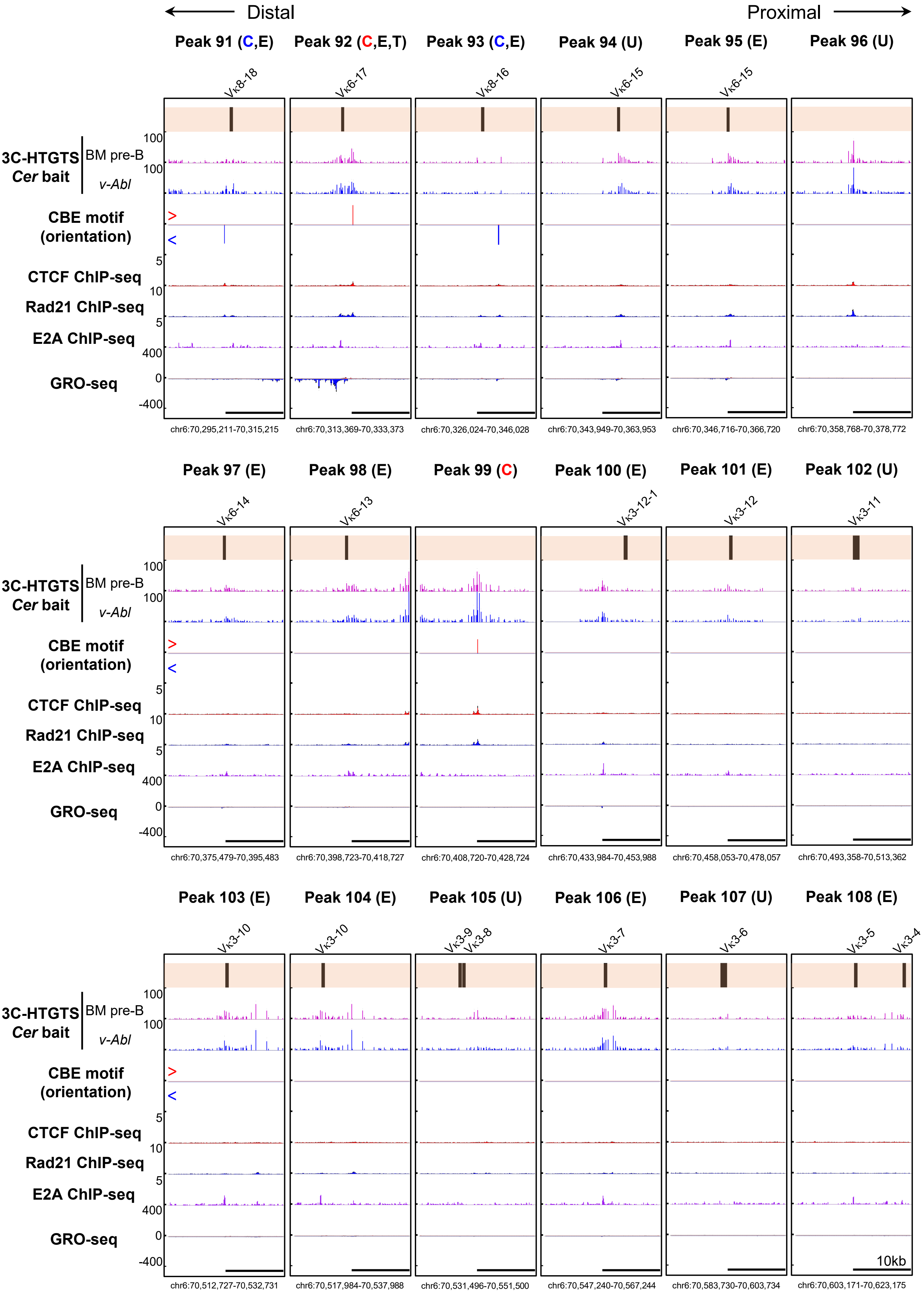

Distal
Proximal
Peak 91 (C,E)
Peak 92 (C,E,T)
Peak 93 (C,E)
Peak 94 (U)
Peak 95 (E)
Peak 96 (U)
Vκ8-18
Vκ6-17
Vκ8-16
Vκ6-15
Vκ6-15
3C-HTGTS
Cer bait
BM pre-B
v-Abl
CBE motif
(orientation)
CTCF ChIP-seq
Rad21 ChIP-seq
E2A ChIP-seq
GRO-seq
chr6:70,295,211-70,315,215
chr6:70,313,369-70,333,373
chr6:70,326,024-70,346,028
chr6:70,343,949-70,363,953
chr6:70,346,716-70,366,720
chr6:70,358,768-70,378,772
Peak 97 (E)
Peak 98 (E)
Peak 99 (C)
Peak 100 (E)
Peak 101 (E)
Peak 102 (U)
Vκ6-14
Vκ6-13
Vκ3-12-1
Vκ3-12
Vκ3-11
chr6:70,375,479-70,395,483
chr6:70,398,723-70,418,727
chr6:70,408,720-70,428,724
chr6:70,433,984-70,453,988
chr6:70,458,053-70,478,057
chr6:70,493,358-70,513,362
Peak 103 (E)
Peak 104 (E)
Peak 105 (U)
Peak 106 (E)
Peak 107 (U)
Peak 108 (E)
Vκ3-10
Vκ3-10
Vκ3-9
Vκ3-8
Vκ3-7
Vκ3-6
Vκ3-5
Vκ3-4
10kb
chr6:70,512,727-70,532,731
chr6:70,517,984-70,537,988
chr6:70,531,496-70,551,500
chr6:70,547,240-70,567,244
chr6:70,583,730-70,603,734
chr6:70,603,171-70,623,175

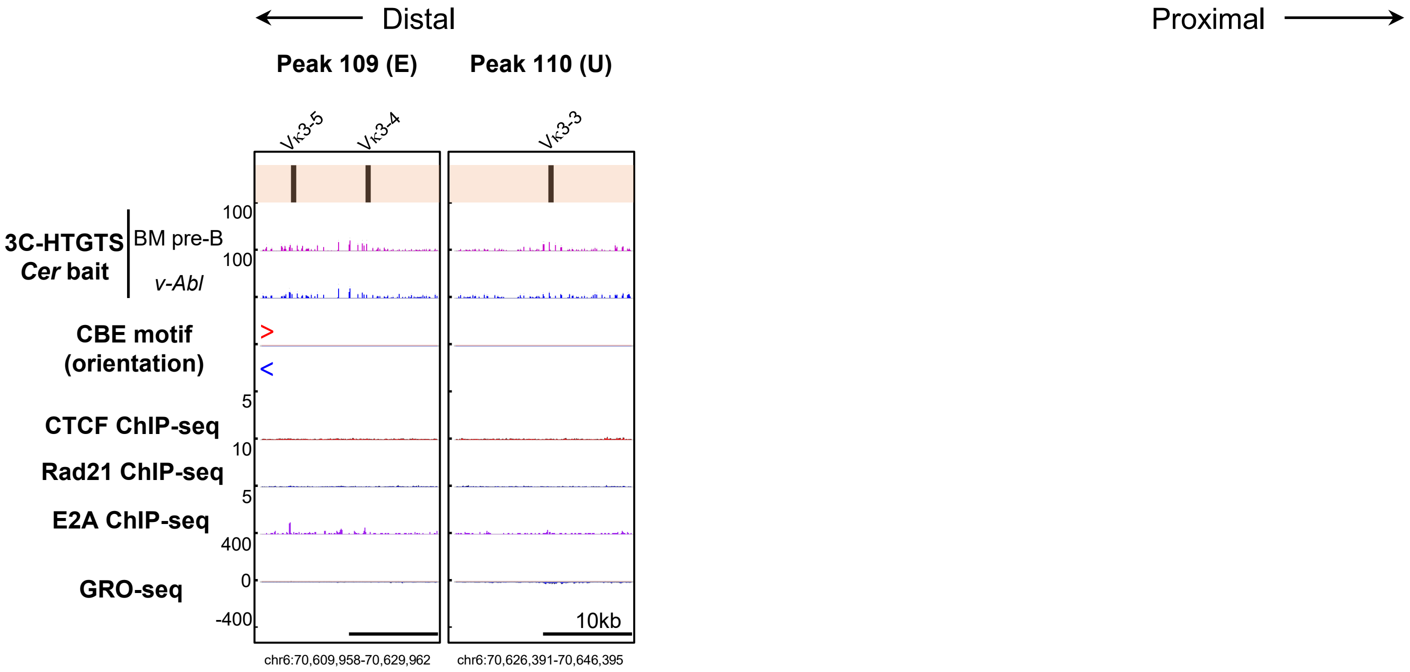
Distal
Proximal
Peak 109 (E)
Peak 110 (U)
Vκ3-5
Vκ3-4
Vκ3-3
3C-HTGTS
Cer bait
BM pre-B
v-Abl
100
100
CBE motif
(orientation)
CTCF ChIP-seq
5
Rad21 ChIP-seq
10
E2A ChIP-seq
5
GRO-seq
400
0
-400
10kb
chr6:70,609,958-70,629,962
chr6:70,626,391-70,646,395
